# Schistosomiasis japonica transmission dynamics: mathematical modeling in guiding One Health approach control strategies

**DOI:** 10.1186/s40249-025-01404-7

**Published:** 2026-01-09

**Authors:** Norvin P. Bansilan, Joaquin M. Prada, Allen Jethro I. Alonte, Martha Elizabeth Betson, Vachel Gay V. Paller, Jomar F. Rabajante

**Affiliations:** 1 Institute of Mathematical Sciences, University of the Philippines Los Baños, Los Baños, Laguna Philippines; 2 School of Veterinary Medicine, University of Surrey, Guildford, UK; 3 Institute of Biological Sciences, University of the Philippines Los Baños, Los Baños, Laguna Philippines

**Keywords:** Schistosomiasis japonica, Mathematical modeling, One Health, Neglected tropical disease, Macroparasite

## Abstract

**Purpose:**

Schistosomiasis (SCH) japonica remains a persistent public health concern in the Philippines despite continuing control efforts. This study aims to examine the transmission dynamics of SCH japonica and evaluate different intervention strategies using a One Health modeling approach, with the goal of supporting feasible control and elimination targets.

**Methods:**

We developed a compartmental mathematical model calibrated using field survey data collected in 2022 from eight endemic barangays in Agusan del Sur and Surigao del Norte. The dataset included SCH prevalence, egg excretion levels in humans and animals quantified through Kato-Katz, modified McMaster, and sedimentation techniques, and household distance to potential transmission sites. Multiple intervention strategies were examined, including human and animal chemotherapy, WaSH (water access, sanitation, and hygiene) adoption, pasture prohibition, vegetation clearing, and snail control. Sensitivity analysis using Partial Rank Correlation Coefficients (PRCC) was performed to identify influential transmission drivers.

**Results:**

The model estimates baseline prevalence at approximately 20% in humans across the study areas. Under medium WaSH adoption, human prevalence is projected to decline to approximately 1.01% by 2030, whereas high WaSH coverage further reduces prevalence to 0.64%. Combining WaSH and pasture prohibition alongside chemotherapy is projected to reduce human prevalence to 0.09% and animal prevalence to 0.10% by 2030. Sensitivity analysis identified snail-to-human transmission rate (PRCC = 0.612) and snail shedding rate (PRCC = 0.607) as the most influential parameters.

**Conclusion:**

Integrated strategies focusing on WaSH, reduced animal exposure, and targeted chemotherapy offer the most effective pathway toward achieving World Health Organization’s (WHO’s) 2030 SCH targets. Implementation should be strengthened through health education, behavioral interventions, mechanization support, and active Local Government Unit (LGU) participation.

**Graphical Abstract:**

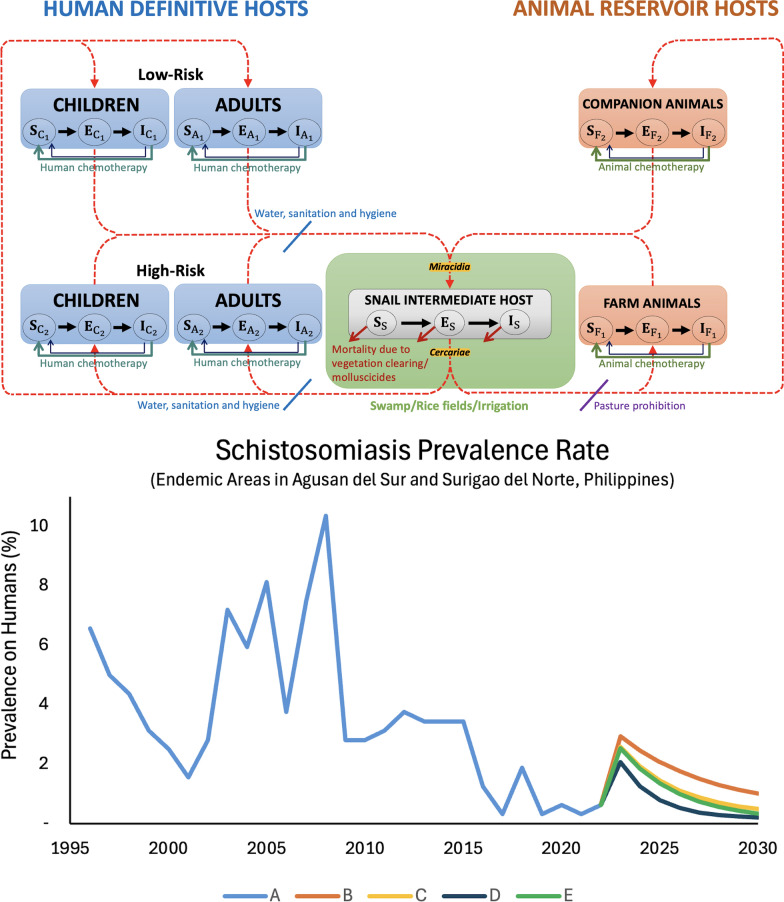

**Supplementary Information:**

The online version contains supplementary material available at 10.1186/s40249-025-01404-7.

## Background

Schistosomiasis (SCH), a neglected tropical disease, is an acute and chronic parasitic ailment caused by blood flukes of the *Schistosoma* genus. In the Philippines, the prevailing schistosome species is *S. japonicum*, characterized as highly zoonotic. More than 40 species of wild and domestic animals, including cats, dogs, cattle, pigs, and rodents, serve as reservoir hosts for *S. japonicum*. Notably, cattle, dogs, cats, and rodents have been identified as potential reservoirs of human infection in the country, with water buffalo being recognized as the primary reservoir [1].

The life cycle of *S. japonicum* intricately involves an infected human definitive host, an animal-reservoir host, and a freshwater snail-intermediate host (Additional file 1: Figure S1). Commencing with the excretion of schistosome eggs through human or animal feces into freshwater, the eggs hatch, releasing miracidia that infect the *Oncomelania hupensis quadrasi* snail. Asexual reproduction unfolds within the snail, progressing through the mother and daughter sporocyst stages. After 4–6 weeks of growth and multiplication, the snail releases numerous infectious larvae, cercariae, into the water. Cercariae, capable of surviving in freshwater for up to 36 h, navigate in search of a suitable definitive or reservoir host. Without finding a host, they perish. Upon encountering a host, infectious cercariae penetrate the skin, shed their forked tails, and transform into juvenile worms known as schistosomula. These schistosomula traverse the body, passing through the lungs, heart, and liver’s portal system, where they mature into adult worms. Adult schistosomes proceed to copulate and migrate to the mesenteric vessels surrounding the small intestine. Female worms lay thousands of eggs daily, with some expelled into the environment through feces, initiating a new life cycle. In contrast, others remain in host tissues, contributing to the manifestation of disease symptoms [2, 3].

SCH japonica imposes a substantial age-specific aggregate disability burden, estimated at 9.80% for children and 18.60% for adults [4]. Manifestations such as hepatomegaly, splenomegaly, portal hypertension, and variceal hemorrhage contribute to malnutrition, anemia, hindered memory development in children, and reduced productivity in adults, collectively perpetuating the cycle of poverty. Neuroschistosomiasis, arising from schistosome egg infection in the brain, has been identified in previous studies, leading to symptoms like headaches and seizures [4]. Complications from untreated SCH japonica infections represent the most prevalent cause of death. The disease predominantly affects impoverished communities unaware of its potential transmission through water sources or unable to avoid contaminated water due to occupational commitments (such as agriculture and fishing), recreational habits (such as swimming), or the absence of access to a reliable source of safe water [4, 5].

The proliferation of *Schistosoma* hinges on completing its entire life cycle. Significantly, intervention strategies can be applied at any developmental stage of the schistosome. Praziquantel, a potent chemotherapeutic drug, effectively reduces adult worm burdens, and while somewhat ineffective against juveniles, it is still the cornerstone intervention for SCH control [6]. Administering praziquantel repeatedly enhances its efficacy, and complementary measures like reducing snail populations by introducing predators, biological competitors, and habitat modifications can be implemented [3]. Although the molluscicide niclosamide stands out as a widely used chemical for snail control, its usage is restricted in the Philippines under the Clean Water Act of 2004 [4]. Despite being non-toxic to humans and animals, it poses a threat to certain freshwater fish and amphibians. Nevertheless, when applied judiciously, niclosamide plays a crucial role in eliminating schistosomes. Additionally, minimizing exposure to contaminated water sites is pivotal in preventing disease transmission. Behavioral modifications, health education initiatives, and enhancements in safe water supply and sanitation practices all contribute to reducing the risk of SCH spread [6].

Since 1965, when Macdonald and Hairston pioneered schistosome models, mathematical modeling has proven to be a compelling approach to understanding the dynamics of SCH [7, 8]. Subsequent models have further enriched our comprehension of the biology and transmission dynamics of SCH, providing crucial insights for the development of prevention programs and public health intervention strategies [9–14].

In the Philippines, Riley et al. [15] conducted a comprehensive study on *S. japonicum* transmission in Leyte, detailing aspects such as infection intensity, age, and sex within the human population. Subsequently, in 2008, Riley et al. expanded their research, creating a mathematical model elucidating *S. japonicum* transmission dynamics among definitive humans, snail-intermediates, and five distinct animal reservoir species in Samar, the Philippines[16]. Meanwhile, Ishikawa et al. [17] devised a model for Bohol Island, considering distinct shedding stages of infected snails based on cercarial output. Their model incorporated seasonal fluctuations and integrated human and snail control measures [17].

Despite ongoing efforts to control and eliminate the disease, SCH japonica continues to pose a significant public health concern in numerous regions across the Philippines. As of 2017, approximately 12 million Filipinos face the threat of SCH japonica, with a direct infection impact on 2.5 million individuals. The Department of Health (DOH) in the Philippines has set a target to reduce the prevalence of high-intensity SCH infections to less than 1 percent by the year 2025 [4, 18].

While existing models and strategies for SCH have been developed, several gaps remain. First, many prior models either treat human infection in isolation or focus narrowly on single interventions, without fully integrating the contribution of animal reservoirs and environmental components that sustain transmission. This is particularly critical in the Philippines, where water buffalo and other domestic and wild animals are well-established reservoirs of *S. japonicum*, yet their roles in transmission control remain underexplored. Second, current control programs rely heavily on mass drug administration (MDA) with praziquantel, which, though effective against adult worms, does not prevent reinfection and fails to address the environmental and animal sources of transmission. Third, few studies have explicitly evaluated how combined interventions interact across the human, animal, and environmental domains, a perspective increasingly recognized as essential by the One Health framework [4].

The One Health approach emphasizes the interconnectedness of human, animal, and environmental health. In the context of schistosomiasis, this approach involves a multi-pronged set of interventions: (1) Human-focused strategies, such as MDA with praziquantel and health education to reduce water contact; (2) Animal-focused strategies, including treatment of reservoir hosts and improved animal husbandry practices to reduce contamination of water sources; and (3) Environmental interventions, such as snail control, modification of snail habitats, provision of safe water sources, and sanitation improvements [2]. By explicitly combining these interventions in a mathematical modeling framework, this study aims to quantify how integrated One Health strategies can accelerate progress toward elimination targets, compared to isolated human-only approaches.

Therefore, this paper constructs a comprehensive mathematical model of *S. japonicum* transmission in the Philippines, incorporating demographic heterogeneity (children vs. adults), domestic and companion animal reservoirs, and vector snails with their larval stages. The model is used to analyze the transmission dynamics and evaluate the effectiveness of One Health-based control strategies. In doing so, this work fills a critical gap in the literature by providing a quantitative assessment of how integrated interventions across humans, animals, and the environment may influence the trajectory toward WHO’s 2030 target of reducing high-intensity schistosomiasis prevalence to below 1%.

## Methods

### Schistosomiasis mathematical model

**Fig. 1:**
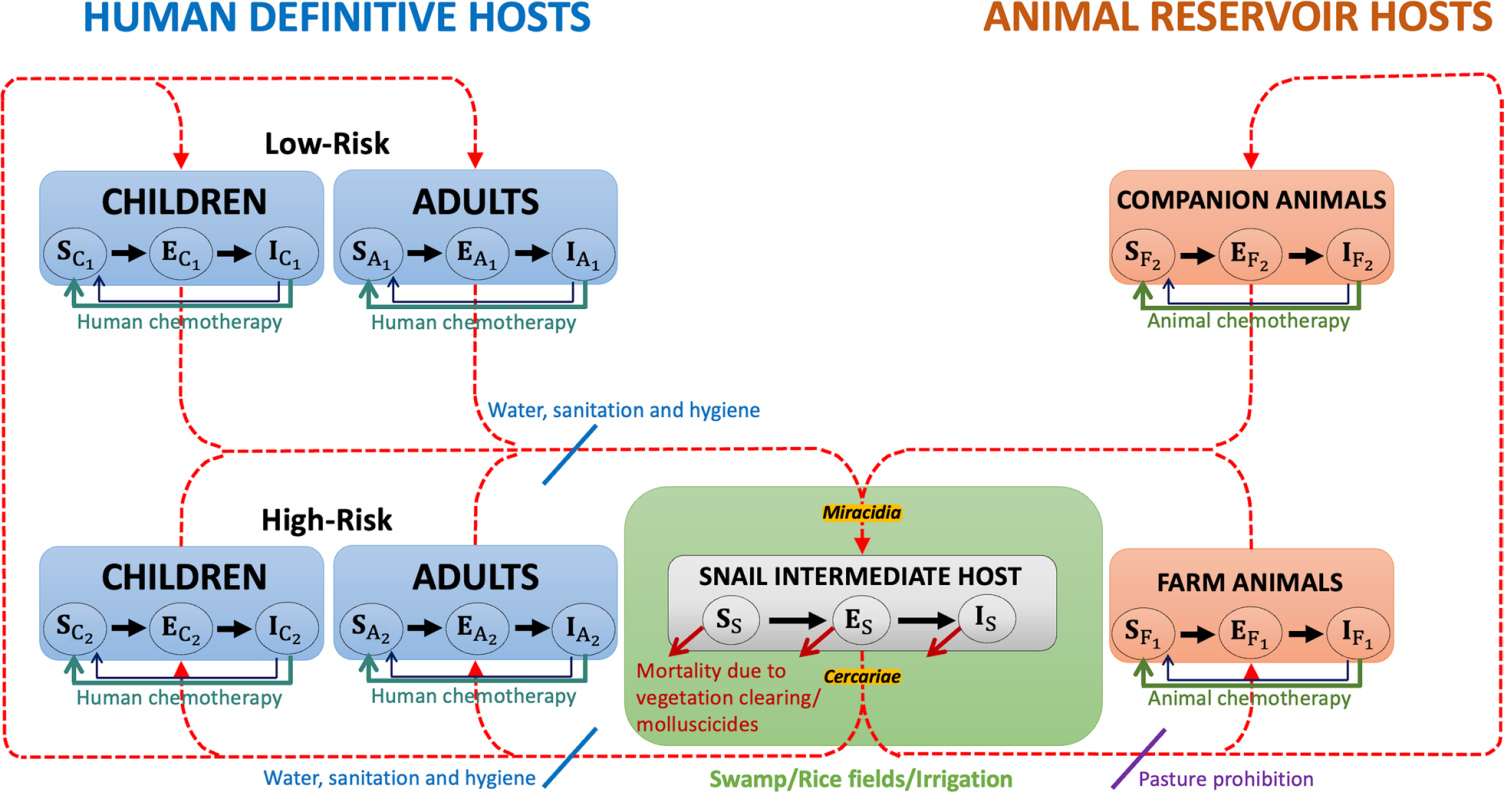
Model diagram of schistosomiasis japonica transmission with interventions. In this diagram, we have the following populations: humans (shaded in blue), animals (in orange), snails (in gray), and two larvae populations (in yellow). The snail habitats, such as swamps, rice fields, or irrigation areas (SRFI), are in green highlights. A further distinction is made between children and adults within the human category. The above compartments denote the low-risk populations, whereas the high-risk populations are delineated in the compartments below. The progression of SCH within each population is illustrated by solid black arrow lines, whereas the transmission of the parasite from humans/animals to snails is indicated by red dashed arrow lines. Green arrows show recovery after human or animal chemotherapy, though reinfection is possible. Blue arrows denote WaSH interventions, which reduce both snail-to-human transmission (by limiting exposure to cercariae) and human-to-snail transmission (by reducing water contamination with miracidia). Purple arrows denote pasture prohibition, preventing snail-to-animal transmission. Snail mortality is indicated from vegetation clearing and/or molluscicide application

The SCH transmission model presented here encompasses various populations, including a human-definitive population, an animal-reservoir population, a freshwater snail-intermediate population, and two larvae populations (miracidia and cercariae) (refer to Fig. 1). In the human population, we differentiate between children (< 20 years old) and adults (≥ 20 years old). Additionally, we differentiate between high and low-risk populations among both humans and animals based on their proximity (for humans) and access (for animals) to snail habitats, such as swamps, rice fields, or irrigation areas (SRFI). Specifically, farm animals like cows, carabaos, and pigs are deemed to have greater exposure to SRFI, while companion animals such as dogs and cats typically have less access to these habitats. Wild reservoirs such as rodents were not included in the model due to lack of site-specific data, but sensitivity analysis was performed to generalize findings to other potential animal hosts outside the default parameterization. Furthermore, we classify human, animal, and snail populations into three epidemiological categories based on their infection status: susceptible, exposed, and infected & infectious. The description of variables for the SCH japonica model is provided in the Additional file 1: Table S1.

We applied logistic growth assumptions to model the introduction of new individuals into the populations. These include newly susceptible children (both high and low risk) and animals (companion and farm). Individuals in the Children category also age into the Adult category (maintaining their respective risk levels), while others are assumed to succumb due to natural death.

Infection occurs when a susceptible human or animal comes into contact with the larvae cercariae in the SRFI, leading the susceptible individual to transition to the exposed population. Following a certain period, this exposed population undergoes a transition to an infected state (and becomes infectious, shedding infective eggs in the environment). While some infected individuals may recover and revert to susceptibility, they otherwise remain infected for life (or until they receive treatment).

We have also incorporated logistic growth into the susceptible snail population, utilizing a fecundity constant for each population category (susceptible, exposed, and infected) [19–21]. Newborn snails are initially classified as susceptible; some perish or transition to the exposed population when in contact with miracidia. The infection of snails is presumed to occur through a combination of miracidia from eggs that had hatched from the feces of children, adults, and animals. The exposed snail population eventually progresses to the infected snail population, where they start shedding cercariae. The subset of infected snails shedding cercariae is assumed to contribute to the cercariae population. Our model posits that once a snail is infected, recovery is not possible, and it inevitably succumbs due to natural death [22]. Furthermore, our model assumes homogeneity in the cercariae population responsible for infecting humans, as well as animals. More details on the model equations are provided in the Additional file 1.

### Epidemiological data and parameter values

Field survey data from the Caraga region of the Philippines, particularly the provinces of Agusan del Sur and Surigao del Norte, obtained in 2022 during the project "Zoonotic Transmission of Intestinal Parasites (ZOOTRIP): Implications for Control and Elimination," was used to parametrize the model. These included SCH prevalence rates, egg excretion levels in humans and animals, and average distances of households to potential transmission sites [23]. Specifically, the data was sourced from eight endemic areas within two provinces. In Agusan del Sur, the sites included: (1) Brgy. Libertad, Bunawan, (2) Brgy. Manat, Trento, (3) Brgy. Taglatawan, Bayugan City, and (4) Brgy. Hawilian, Esperanza. Meanwhile, in Surigao del Norte, the study covered: (5) Brgy. San Isidro, Mainit, (6) Brgy. Buhing Calipay and Brgy. Del Pilar, San Isidro, (7) Brgy. Daywan, Claver, (8) Brgy. San Isidro, Gigaquit. Prevalence in human and animal populations was estimated through the detection of schistosome eggs in fecal samples. The Kato-Katz technique quantifies the number of eggs per gram of feces (epg) excreted by infected children and adults. Meanwhile, modified McMaster, simple sedimentation, and sucrose flotation techniques are employed to determine the epg excreted by infected animals.

In our model, to remain conservative, we take the highest epg excreted by infected children, adults, and animals in the study area (i.e., 269 epg, 108 epg, and 62 epg, respectively) [24]. Humans excrete 200 g of feces each day on average [25]. Thus, we assumed that 269 × 200 = 53,800 eggs, 108 × 200 = 21,600, and 62 × 200 = 12,400 eggs are excreted per day by infected children, adults, and animals, respectively. These expelled eggs are assumed to eventually reach the water and hatch. The probability of eggs reaching water and hatching is scaled for each population based on their proximity and access to snail habitats. In low-risk populations, this probability is reduced by half compared to high-risk populations [26]. This relatively high value was selected to represent an extreme yet realistic scenario, which enables evaluation of intervention effects under conditions of maximal transmission pressure. Additionally, the study records the average distance (in kilometers) between snail sites and households, crucial for assessing the proximity of the population to infection sites and distinguishing between high and low-risk groups. Households within or equal to 0.2 km from snail sites are categorized as high-risk, while those situated beyond 0.2 km are considered low-risk (Additional file 1: Table S2 and Table S3). The 0.2 km threshold reflects the typical dispersal range of snails and the flow of contaminated water carrying cercariae. People living or working within this distance, especially in rice fields, are more likely to come into contact with infected water. Using this range helps target high-risk areas more effectively for SCH control.

Annual SCH prevalence data (1996–2022) for Agusan del Sur and Surigao del Norte were obtained from the Philippine Department of Health website. We used these data to estimate the prevalence rate of the observed endemic areas in the provinces [27, 28]. Due to the limited data available and the lack of detailed intervention information in that period, we assumed minimal control measures were in place during this period (e.g., annual human deworming treatment at a low coverage). We chose 2008 as the baseline for fitting the transmission rates from snail to human and animal hosts, as that was the time point with the highest reported prevalence. We further assumed that the transmission rates of high-risk populations were ten times higher than the transmission rates of the low-risk populations. On the other hand, SCH field data from the ZOOTRIP project were used to estimate the prevalence across various populations and to simulate different intervention scenarios, as explained in the following section.

The demography parameters for both human and animal populations, including birth and death rates, recovery rates, and latent periods, were collated from the literature [29–42]. Similarly, online references were used for the biology and epidemiology parameters of snail and schistosome larvae [19, 43–45]. Since there was no acquired prevalence data for animals, the calculated total maximum number of farm and companion animals was used to obtain the prevalence rate of animal populations. For a detailed derivation of the parameters utilized in the model, refer to the Additional file 1: Parameter Values and Table S4.

### Model interventions

In our model, we incorporated several WHO-recommended interventions aimed at eliminating the disease as a public health problem, including human chemotherapy, animal chemotherapy, WaSH, pasture prohibition, and snail control (via vegetation clearing and/or molluscicides). As depicted in Fig. 1, infected humans will recover following treatment, but subsequently become susceptible to reinfection. Similarly, infected animals are anticipated to recover after chemotherapy, returning to a susceptible state. In the Philippine context, particularly in resource-limited rural communities, households and workplaces such as rice farming are frequently located in close proximity to infected water sources. As a result, reinfection remains highly likely. Additionally, WaSH is presumed to act as a preventive measure, reducing both the transmission of infection from snails to humans and from humans to snails. Meanwhile, pasture prohibition is assumed to serve as a preventive barrier, mitigating the transmission of infection from snails to animals. Furthermore, ongoing snail control through vegetation clearing and/or molluscicides is assumed to target both uninfected and infected snails.

To evaluate the impact of these interventions, we conducted simulations using our model in RStudio 2024.09.0 Build 375 (Posit Software, PBC, Boston, MA, USA), applying the discretized step method with a time increment ($\Delta t$) of 0.01 per day over eight years. This period, starting from 2023 to 2030, was selected to align with the WHO’s 2030 target of reducing SCH prevalence rates to below 1%. To estimate the prevalence of the different populations, we used the 2022 field data from the ZOOTRIP project. The interventions were analyzed at three implementation levels: baseline (0%), medium (50%), and high (100%), which represent assumed proportional effects of the interventions on relevant biological processes (e.g., recovery, transmission, snail mortality) throughout the simulation period. Here, 0% corresponds to the baseline scenario with no additional intervention beyond existing control measures already in place at the study site, 50% represents a 1.5-fold increase from baseline, and 100% represents a twofold increase. These levels were chosen for simplicity to capture key implementation scenarios and to illustrate the range of potential outcomes. Each intervention, such as repeated chemotherapy for humans and animals, WaSH practices, pasture prohibition, and snail control, was assumed to be implemented consistently daily throughout the eight-year simulation. Specifically, WaSH practices and pasture prohibition were assumed to be practiced daily by the corresponding proportion of households, while interventions like snail control and chemotherapy, once initiated, were considered continuously active and effective throughout the simulation period (Table 1).

**Table 1:** Assumed proportional effects of interventions at varying implementation levels

Interventions	Without (0%)	Medium (50%)	High (100%)
Human chemotherapy	No increase in recovery rate among infected humans	50% increase in recovery rate from baseline	Maximum possible increase in recovery rate
Animal chemotherapy	No increase in recovery rate among infected animals	50% increase in recovery rate from baseline	Maximum possible increase in recovery rate
WaSH	No reduction in transmission or contamination	50% reduction in egg deposition and exposure to cercariae	Maximum reduction in egg deposition and exposure
Pasture prohibition	No reduction in animal exposure to contaminated water	50% reduction in exposure to highrisk areas	Full reduction in exposure to highrisk areas
Snail control	No increase in snail mortality	Partial vegetation clearing to moderately increase snail mortality	Vegetation clearing with molluscicide to maximize snail mortality

*WaSH* Water access, sanitation, and hygiene

Human chemotherapy increases the recovery rate among infected humans, while animal chemotherapy increases the recovery rate among infected animals [32]. The implementation levels (0%, 50%, 100%) scale these recovery rates proportionally; for example, 50% implementation of human chemotherapy corresponds to a 50% increase from the baseline recovery rate toward its maximum possible value. On the other hand, WaSH interventions reduce both the likelihood of infected individuals contaminating the environment (e.g., through egg deposition) and the probability of susceptible individuals becoming infected (e.g., through contact with cercariae in contaminated water) [19]. In the model, a 50% implementation of WaSH corresponds to a 50% reduction in both human egg deposition into the environment and human contact with cercariae. Similarly, pasture prohibition reduces the exposure of animals to contaminated water bodies or snail-infested environments, thus lowering the likelihood of animal infection. Implementation levels reflect the proportion of households consistently restricting animal access to high-risk areas. Finally, snail control increases the mortality of intermediate host snails and reduces their habitat suitability. Moderate levels of implementation (50%) reflect partial vegetation clearing, while full implementation (100%) combines vegetation clearing with molluscicide application to maximize snail mortality [1].

To explore One Health control strategies for SCH, we simulated all possible combinations of five individual interventions, human chemotherapy, animal chemotherapy, WaSH, pasture prohibition, and snail control, each at three implementation levels (0%, 50%, 100%). This yielded a total of 243 unique intervention combinations (3^5^). We assessed the effect of each intervention by fixing its level and allowing all other interventions to vary across their possible combinations (Additional file 1: Model Interventions). For example, in human chemotherapy, the distribution of prevalence outcomes across 81 combinations without human chemotherapy, 81 combinations with medium human chemotherapy, and 81 combinations with high human chemotherapy. While the other four interventions (animal chemotherapy, WaSH, pasture prohibition, and snail control) vary across all levels. This approach isolates the effect of a single intervention while accounting for interaction with other control measures. The same logic applies to animal chemotherapy, WaSH, pasture prohibition, and snail control, which display the impact of fixing other interventions while allowing the rest to vary (Additional file 1: Table S5).

Furthermore, we selected several simulated combinations and plotted their projected prevalence rates for the next eight years (2023–2030). We have the following: historical data from 1996 to 2022 (A), with medium WaSH only (B), with medium human chemotherapy and medium WaSH only (C), with high human chemotherapy and medium WaSH only (D), and with medium human, medium WaSH, and vegetation clearing (E). As observed, all presented combinations demonstrate at least a medium level of WaSH implementation. According to the 2023 DOH annual report, the Caraga region reported that 93.47% of households had access to basic water supply, while 85.39% had access to basic sanitation facilities [27].

### Sensitivity analysis

To assess the relative influence of model parameters on infection dynamics, a global sensitivity analysis was performed. Both initial values and parameter estimates were perturbed by an order of magnitude of 10 to capture potential uncertainty in the system. A total of 2000 simulation runs were carried out using Latin Hypercube Sampling (LHS) to efficiently explore the parameter space.

The sensitivity of each parameter was quantified using PRCC, which measure the monotonic relationship between parameters and model outcomes while controlling for the effects of other parameters. PRCC values close to +1 or −1 indicate strong positive or negative influence, respectively, whereas values near zero indicate negligible effect.

## Results

### Individual interventions

**Fig. 2:**
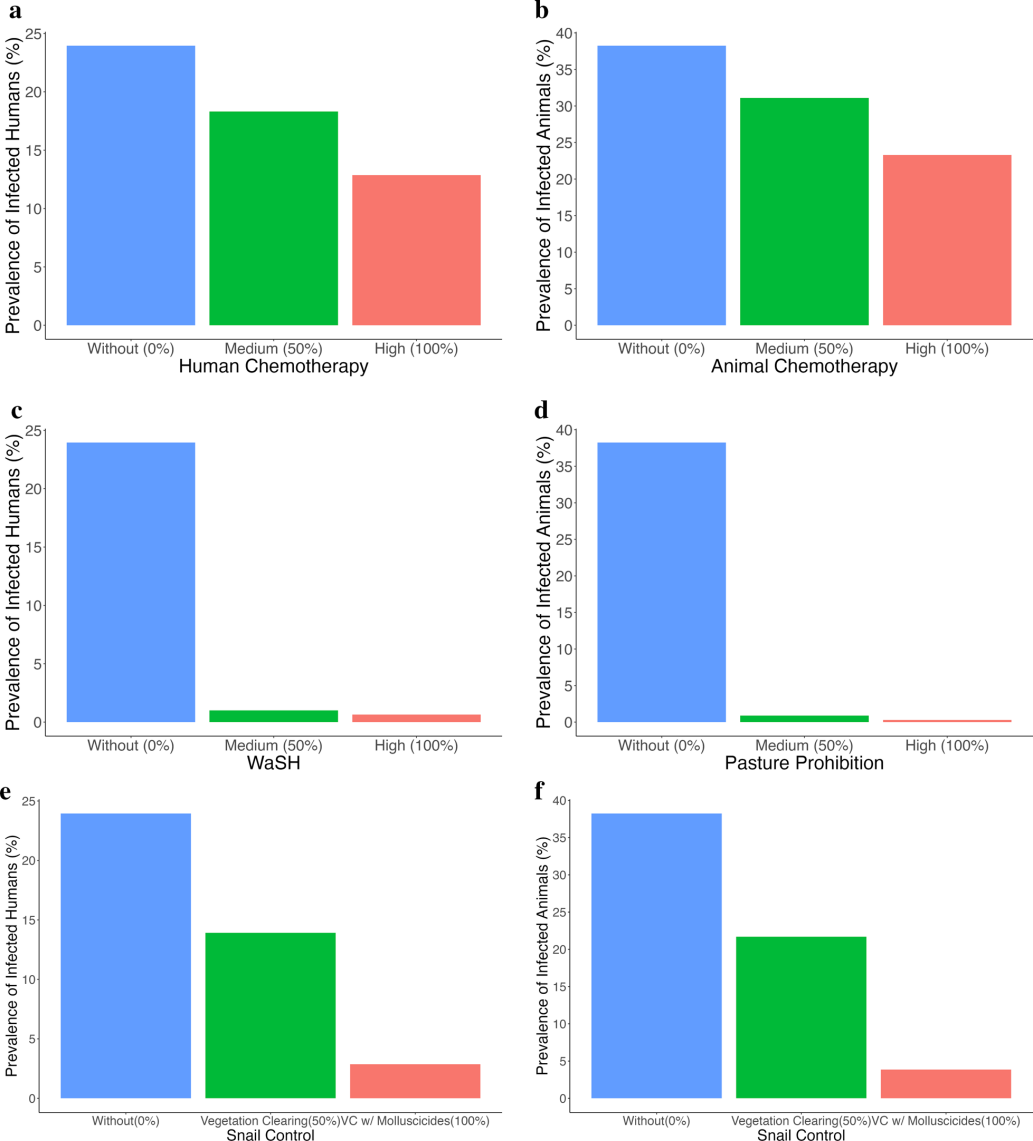
Individual interventions: human chemotherapy, animal chemotherapy, WaSH, pasture prohibition, and snail control. The x-axis denote the individual interventions with different implementations—Without (blue), Medium (green), and High (red). While y-axis denote the estimated prevalence rate of infected populations (in percent) after eight years (in 2030) in the endemic areas of Agusan del Sur and Surigao del Norte. In these simulations, only one intervention is implemented, e.g., in (a), only human chemotherapy (50% or 100%) is applied without animal chemotherapy (0%), WaSH (0%), pasture prohibition (0%), and snail control (0%). *WaSH* Water access, sanitation, and hygiene

Figure 2 shows the projected prevalence rates (in percent) of infected populations in 2030, the end of the eight-year simulation period (2022–2030), under different individual intervention scenarios in the endemic areas of Agusan del Sur and Surigao del Norte. The "without intervention" scenario represents the current baseline conditions, assuming no additional control measures are implemented. Under this scenario, the projected prevalence in 2030 is 23.95% for humans and 38.24% for animals.

Implementing medium human chemotherapy reduces the prevalence of infected humans to 18.31%, while high human chemotherapy further lowers it to 12.86% (Fig. 2a). In the case of animals, medium animal chemotherapy results in a 31.10% prevalence rate, whereas high animal chemotherapy decreases it to 23.30% (Fig. 2b).

Figure 2c highlights the impact of WaSH interventions on human infections. Medium WaSH reduces prevalence to 1.01%, while high WaSH brings it down further to 0.64% by 2030. Similarly, pasture prohibition demonstrates a strong effect in reducing animal infections, with medium implementation achieving a prevalence of 0.90%, and high implementation reducing it to 0.30% (Fig. 2d).

As shown in Fig. 2e, snail control through vegetation clearing alone results in a 13.92% human prevalence and 21.70% in animals. When combined with molluscicide application, the intervention achieves a more substantial reduction, 2.87% in humans and 3.85% in animals (Fig. 2f).

The following results illustrate the projected active cases of infected populations under individual intervention scenarios over the eight years in the endemic areas of Agusan del Sur and Surigao del Norte. Medium and high human chemotherapy exert a moderate impact on reducing human infections, see Additional file 1: Figure S2. Notably, during the early years of implementation, there is a rapid increase in infections among high-risk populations, while low-risk populations show a more gradual rise.

Similarly, medium and high animal chemotherapy have limited effects on reducing infection in both farm and companion animals, see Additional file 1: Figure S3. A comparable early surge in cases is observed in farm animals relative to companion animals.

In contrast, WaSH interventions (Additional file 1: Figure S4) have a substantial impact in reducing infection prevalence among both low-risk and high-risk humans over the simulation period. Likewise, pasture prohibition leads to significant reductions in infection rates among farm and companion animals, as shown in Additional file 1: Figure S5.

Finally, vegetation clearing alone results in a noticeable decline in infections across both risk groups of humans and animals, Additional file 1: Figure S6. When molluscicide application is added, the impact becomes more pronounced, yielding a remarkable reduction in infected populations across all host types and risk groups.

### One Health approach strategies

**Fig. 3:**
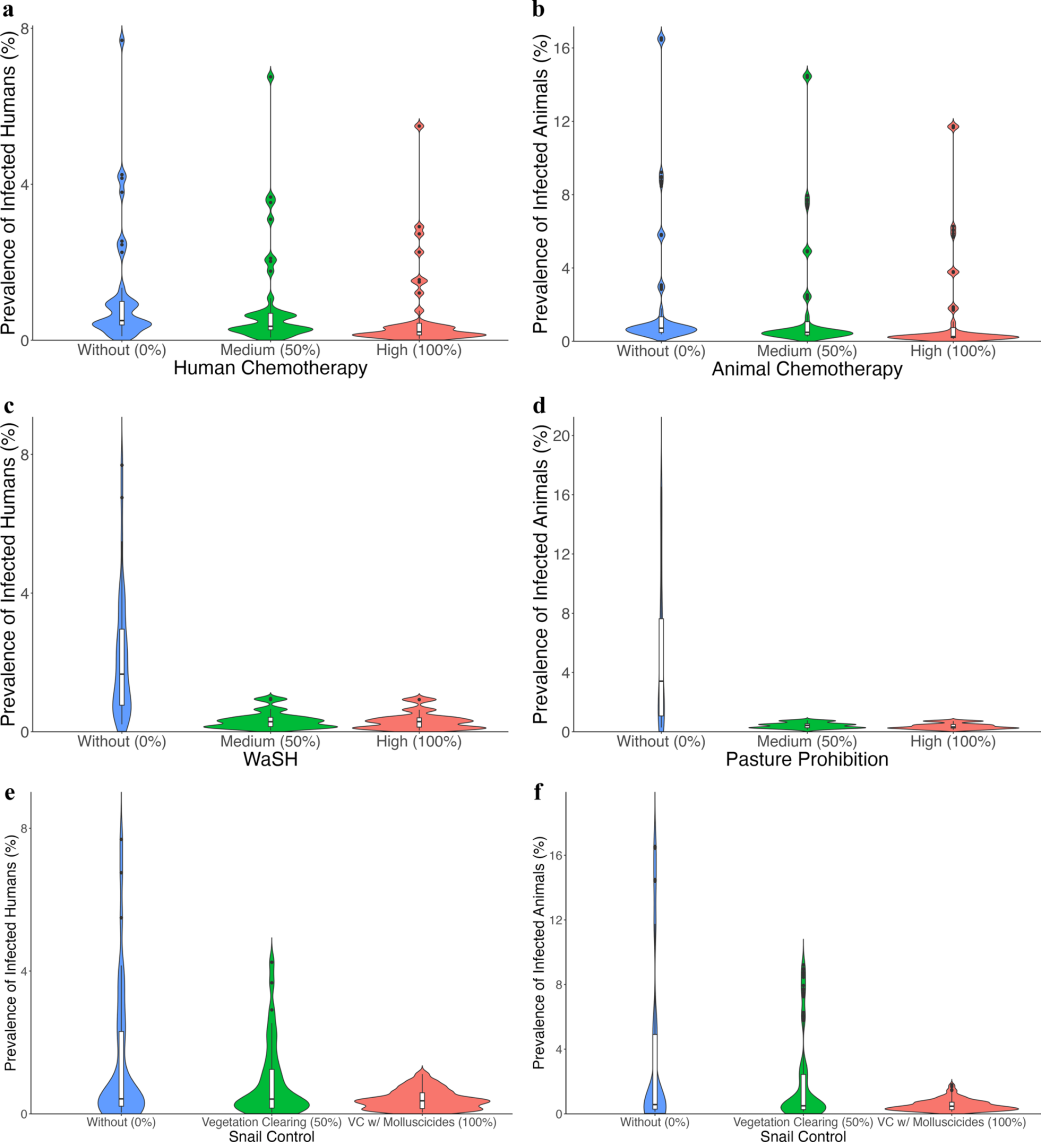
One Health approach: combinations of interventions—human chemotherapy, animal chemotherapy, WaSH, pasture prohibition, and snail control. The x-axis denote the combinations of interventions with different implementations—Without (blue), Medium (green), and High (red). While y-axis denote the prevalence rate of infected populations (in percent) after three years in the endemic areas of Agusan del Sur and Surigao del Norte. In the simulations, each panel illustrates the effect of one fixed intervention, while the remaining four interventions vary across all possible combinations. For example, in **a**, human chemotherapy is fixed at three levels, without (0%), medium (50%), and high (100%), while animal chemotherapy, WaSH, pasture prohibition, and snail control are varied across their full implementation range. The resulting distributions of prevalence are shown for each fixed level of the target intervention. *WaSH* Water access, sanitation, and hygiene

We explored One Health control strategies for SCH by simulating various combinations of intervention implementations. As shown in Fig. 3, medium-level human and animal chemotherapy result in minimal changes in the prevalence of infected populations (Fig. 3a and b), with more noticeable reductions occurring only at high implementation levels.

In contrast, both medium and high levels of WaSH and pasture prohibition lead to similarly significant reductions in human and animal infections, respectively (Fig. 3c and d).

Furthermore, vegetation clearing alone produces a moderate reduction in infections among both humans and animals (Fig. 3e and f). However, when vegetation clearing is combined with molluscicides, a substantially greater decrease in prevalence is observed, highlighting the enhanced effectiveness of integrated snail control strategies.

The following results illustrate selected One Health strategies employed for controlling SCH. After eight years of implementation of a combination of high human chemotherapy, medium WaSH, and vegetation clearing, Additional file 1: Figure S7, a substantial reduction is predicted in the active infection prevalence among low-risk and high-risk human populations, reaching 0.09%. However, infection levels among farm and companion animals continue to rise, reaching a prevalence of 19.70%, highlighting the limitations of human-targeted interventions alone.

In contrast, a more comprehensive One Health approach, incorporating high human and animal chemotherapy, medium WaSH and pasture prohibition, and vegetation clearing, Additional file 1: Figure S8, leads to significant reductions across all populations after eight years, with human infection prevalence again dropping to 0.09%, and animal infection prevalence reduced dramatically to 0.10%.

**Fig. 4:**
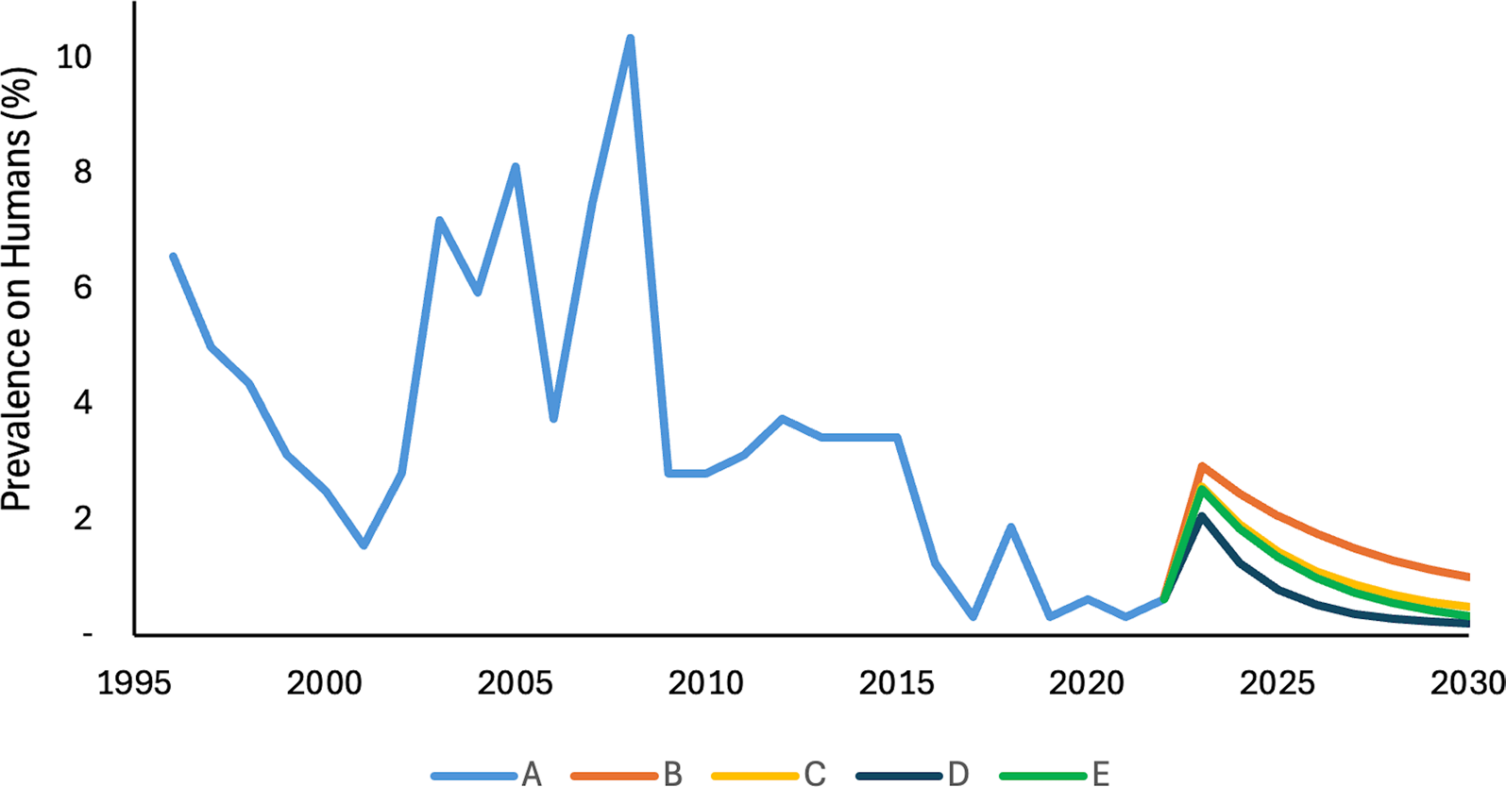
Schistosomiasis prevalence rate (in percent) on humans in the endemic areas of Agusan del Sur and Surigao del Norte, Philippines from 1996 to 2030. The diagram shows the historical data of prevalence from 1996 to 2022 and simulated prevalence after eight years with different scenarios: **A** historical data; **B** with medium WaSH only, **C** with medium human chemotherapy and medium WaSH only; **D** with high human chemotherapy and medium WaSH only; and **E** with medium human chemotherapy, medium WaSH, and vegetation clearing. *WaSH* Water access, sanitation, and hygiene

Furthermore, Fig. 4 illustrates the SCH prevalence rate in humans within the endemic areas of Agusan del Sur and Surigao del Norte, Philippines, spanning from 1996 to 2022 (line A). The highest recorded prevalence during this period was 10.31% in 2008. For the subsequent eight years (2023–2030), we simulated various intervention combinations to project future trends. By 2030, the prevalence under Implementation B is projected to decline to 0.94%, while Implementation C is expected to reach 0.63%. Notably, both Implementations D and E are projected to reduce prevalence further to 0.31%. The sharp increase in prevalence observed in 2023 reflects the modeling assumption that transmission rates were fitted using the peak prevalence recorded in 2008, providing a conservative baseline for the simulation.

In barangays within endemic areas, medium-level WaSH implementation alone is insufficient to substantially reduce SCH prevalence. Additional file 1: Table S6 presents human prevalence rates for endemic barangays in Agusan del Sur and Surigao del Norte under a combined intervention of medium WaSH and high human chemotherapy, using 2022 field data as a baseline and projecting outcomes for 2026 and 2030. As shown, six out of eight endemic areas are still projected to have prevalence rates exceeding 1% by 2030. To improve outcomes, Table 2 recommends a more intensive strategy combining high WaSH, high human chemotherapy, and vegetation clearing. This integrated approach reduces prevalence to below 1% in all areas except for Barangay Buhing Calipay/Del Pilar, San Isidro, Surigao del Norte, where the projected prevalence remains at 1.29% by 2030. This residual burden may be attributed to socioeconomic challenges, including high poverty incidence in the area. It is also important to note that only human prevalence data were presented, as limited animal SCH prevalence data were available.

## Discussion

Numerical simulations were conducted to determine the effectiveness of several WHO-recommended interventions in eliminating the disease. Based on our results, in the absence of any intervention, prevalence in humans would be expected to rise to 23.95% in this region. With medium and high human chemotherapy, the prevalence rates decrease to 18.31% and 12.86%, respectively. Our findings suggest that, even with repeated administration, human chemotherapy has only a moderate effect on the infected human population. This observation may be attributed to the continuous exposure of humans to contaminated water, resulting in reinfection. Additionally, results show a rapid increase in infected cases in high-risk populations compared to low-risk populations. This discrepancy could be attributed to the proximity of each population to the SRFI. It implies that infection spreads more rapidly in households near contaminated water than in those far away.

Similarly, the findings underscore that animal chemotherapy alone has a limited impact on the infected animal population, even with repeated administrations. This phenomenon may be attributed to animals continually being exposed to contaminated water, leading to reinfection. Moreover, infection rates rise more rapidly in farm animals than in companion animals, primarily due to greater exposure to contaminated environments such as SRFI.

Meanwhile, with medium and high WaSH, the prevalence in 2030 is projected to decrease to 1.01% and 0.64%, respectively. The findings strongly suggest that the continuous practice of WaSH has a noteworthy effect in reducing the infected human population, potentially by interrupting the transmission from snail to human and human to snail. Also, these findings suggest that WaSH reduces a higher number of infected humans than human chemotherapy. Notably, both medium and high WaSH implementations exhibit nearly identical reductions in prevalence, suggesting that medium coverage represents a practical benefit inflection point, beyond which additional investment yields only marginal improvements. This implies that achieving moderate but sustained coverage may already capture most of the achievable epidemiological benefit, making it more cost-efficient than striving for unrealistic full compliance.

However, the effectiveness of WaSH depends on the completeness of its components. For example, if sanitation is lacking, such as the absence of functional latrines, then contamination may persist despite other WaSH efforts, ultimately limiting the intervention’s impact. This underscores the importance of comprehensive and well-observed WaSH implementation, where all key components, water, sanitation, and hygiene, are addressed together. Strengthening WaSH programs through behavioral change, health education, and LGU support can greatly enhance long-term effectiveness in endemic communities. Practical measures include building community latrines in high-prevalence areas, installing safe washing stations near rice fields and irrigation canals, and promoting household water filtration, steps that directly reduce snail habitat contamination and daily exposure risks.

The findings also indicate that the sustained implementation of pasture prohibition significantly reduces infection among animals, likely by disrupting the transmission pathway from snails to animal hosts. Moreover, pasture prohibition results in a greater reduction in the number of infected animals compared to animal chemotherapy. Similar to WaSH, both medium and high pasture prohibition scenarios yield nearly identical reductions in infection levels, again suggesting a benefit inflection point at moderate compliance. This supports prioritizing medium adoption levels, which are more feasible for communities and yet already provide substantial epidemiological benefit. Pasture prohibition can be made feasible with mechanization support, such as providing tractors to reduce reliance on working animals. Adoption challenges may be mitigated through LGU assistance in the form of resources, incentives, and sustained support. Complementary measures include advancing livestock vaccination research, especially for bovines, the main reservoir in the Philippines, and enforcing pasture management regulations to limit grazing near snail habitats.

Our results show that preventive environmental measures lead to greater reductions in schistosomiasis cases than relying on chemotherapy alone. Interrupting transmission from snails to humans or animals produces a more substantial decline in infections, reflecting the complex dynamics of the parasite’s life cycle. Even individuals and animals treated through chemotherapy remain susceptible to reinfection, as ongoing water activities, inadequate water supply, and poor sanitation sustain miracidia, infected snails, and cercariae in the environment.

While mass drug administration remains the primary control strategy, long-term success requires integrating WaSH measures to prevent reinfection and reduce contamination of snail habitats. However, achieving 100% WaSH coverage or strict pasture prohibition is unrealistic in resource-limited endemic settings. A phased approach, gradually raising compliance from moderate to high through community engagement and LGU support, can still significantly reduce transmission. Once prevalence is driven to low levels, maintaining moderate compliance may be sufficient to prevent resurgence, as the parasite’s life cycle becomes harder to sustain [1].

On snail control implementation, the prevalence rate decreased to 13.92% with vegetation clearing alone, while combining it with molluscicides further reduced prevalence to 2.87% (Fig. 2). Our results show that snail control using vegetation clearing alongside molluscicides yields larger reductions than vegetation clearing alone. However, since molluscicides are restricted in the Philippines under the Clean Water Act of 2004, sustainable alternatives are needed. Potential strategies include vegetation clearing and habitat modification, such as periodic drainage of irrigation canals, proper canal lining, and water flow regulation, to disrupt snail breeding habitats. In addition, biological control strategies may involve enhancing or introducing natural snail predators such as ducks, frogs, or certain fish species that graze on snails, providing an ecologically stable form of population suppression. These measures offer more sustainable and environmentally responsible options within a One Health framework, particularly in agricultural ecosystems where chemical-based approaches are difficult to justify.

**Table 2:** Combination of high WaSH, high human chemotherapy, and vegetation clearing implementation

Province	Municipality	Barangay	Prevalence Rate (%)		
			2022	2026	2030
			(Field data)	(Predicted)	(Predicted)
Agusan del Sur	Bunawan	Libertad	3.27	1.90	0.20
	Trento	Manat	6.11	3.56	0.37
	Bayugan City	Taglatawan	1.77	1.03	0.11
	Esperanza	Hawilian	8.15	4.74	0.50
Surigao del Norte	Mainit	San Isidro	12.37	7.20	0.75
	San Isidro	Buhing Calipay/Del Pilar	21.27	12.37	1.29
	Claver	Daywan	7.35	4.28	0.45
	Gigaquit	San Isidro	5.56	3.24	0.34

An environmental approach targeting transmission interruption by eradicating vector snails has proven to be an effective intervention for eliminating SCH. According to Sokolow et al. [46], snail control is an effective strategy in reducing SCH prevalence. Despite evidence supporting the long-term decrease and elimination of the disease through snail control, ongoing SCH management efforts prioritize MDA with praziquantel over snail control. Combining drug-based initiatives with low-cost snail control emerges as a more effective and comprehensive approach for SCH elimination [46].

However, using molluscicides in snail control raises concerns about environmental pollution and unintended ecological impacts. The amphibious nature of *Oncomelania* spp*.* snails, responsible for transmitting *S. japonicum*, poses a challenge as molluscicides are less effective against them. The avoidance behavior of these snails, such as burrowing or leaving the water to escape chemicals, necessitates repeated applications. Considering the vast areas of swamps, rice fields, or irrigations, coupled with the rapid reproduction of snails in freshwater, implementing snail control with molluscicides may be challenging, environmentally hazardous, and expensive [47]. Despite the efficacy of snail control, current control programs predominantly emphasize MDA, relegating costly snail control measures and environmental modifications to supplementary roles [4].

Regarding the combination of interventions, the results suggest that optimizing One Health strategies requires administering higher levels of both human and animal chemotherapy. In contrast, moderate implementation levels of WaSH and pasture prohibition appear sufficient when combined with interventions targeting both host populations. This dual approach is crucial, as focusing on either humans or animals alone is insufficient to interrupt SCH transmission. Given the parasite’s life cycle, SCH can persist in animals even without human hosts, and vice versa. Therefore, effectively reducing infection prevalence in both populations necessitates addressing all major reservoirs of infection through a truly integrated One Health framework.

However, in endemic areas, the findings highlight that high-level WaSH implementation is necessary to achieve the DOH and WHO’s 2020 target of reducing prevalence rates to less than 1%. While prevalence rates may appear low from a macro perspective, such as at the regional or provincial level, a closer, micro-level analysis at the barangay level reveals significantly higher prevalence rates. This disparity underscores why the disease continues to be classified as a neglected tropical disease.

It is important to acknowledge some simplifying biological assumptions in the model, such as uniform intervention adherence and static environmental conditions. Despite these conservative assumptions, the model still projects that achieving WHO elimination targets may require full (100%) implementation of key interventions, an unrealistically high threshold in many real-world settings. This highlights the practical and logistical challenges of reaching the WHO 2030 goals for SCH particularly in resource-limited or logistically complex environments.

Furthermore, in alignment with the Clean Water Act of 2004, prioritizing vegetation clearance over molluscicides is recommended for a more cohesive One Health approach.

The results of our sensitivity analysis demonstrate that SCH transmission is most sensitive to parameters linked to snail-to-human transmission, snail population capacity, and cercarial survival in the environment. In particular, the snail-to-human transmission rate (PRCC = 0.612) and snail shedding rate (PRCC = 0.607) emerged as the strongest positive drivers of prevalence, underscoring that increased human exposure to cercariae and sustained snail abundance are central to transmission. Conversely, the human recovery rate (PRCC = −0.493) and cercarial death rate (PRCC = −0.593) showed strong negative effects, highlighting that treatment-related increases in recovery and environmental conditions that reduce cercarial survival effectively suppress transmission. These findings emphasize the importance of minimizing water contact and exposure, managing snail populations, and strengthening environmental interventions such as vegetation clearing or snail control strategies.

Taken together, these findings underscore the necessity of integrated One Health strategies. While human chemotherapy remains effective in reducing worm burdens, sustained control cannot be achieved through MDA alone. Instead, long-term success depends on complementary interventions that lower snail abundance, reduce cercarial production, improve recovery in humans and animals, and limit environmental contamination. Our results emphasize that targeting multiple stages of the parasite’s life cycle across human, animal, and environmental domains is essential for durable control.

This integrated perspective directly supports the DOH and WHO goals of reducing high-intensity schistosomiasis prevalence to below 1%. More broadly, it reinforces that achieving elimination as a public health problem requires moving beyond human-only interventions toward coordinated One Health approaches that simultaneously address transmission at its ecological, veterinary, and community health roots. Compared to earlier models, our approach provides novel insights by explicitly incorporating WaSH, pasture prohibition, and other One Health interventions, as well as identifying key transmission drivers through sensitivity analysis. These additions make our model particularly insightful for guiding integrated and sustainable policy strategies. Moreover, the model framework is transferable to other S. japonicum endemic areas, as its structure and key transmission parameters are broadly generalizable. The parameters identified as most influential in the sensitivity analysis can serve as a basis for prioritizing interventions across different settings.

Although cost data remain limited, meaningful insights emerge regarding the relative costs and impacts of various SCH interventions, providing a valuable foundation for future planning. For instance, human chemotherapy is generally less expensive and easier to deploy at scale compared to WaSH interventions. However, WaSH measures tend to result in a more substantial reduction in human infection rates, highlighting the trade-off between short-term affordability and long-term effectiveness [48–50].

Similarly, in the context of animal interventions, animal chemotherapy is generally more affordable and logistically simpler to implement than pasture prohibition. However, its availability may pose a challenge in the Philippine setting. On the other hand, pasture prohibition leads to a more substantial reduction in animal infections by directly targeting environmental transmission pathways. While promising in terms of disease control, this strategy may also impact farming practices, particularly for communities that rely on animals for agricultural work. These considerations highlight the need to balance feasibility, effectiveness, and socio-economic impacts when designing sustainable animal health interventions.

Taken together, these insights suggest that while lower-cost interventions can deliver immediate benefits, those that address environmental transmission may offer more sustainable outcomes in the long run. The outcomes gleaned from our study provide important guidance on factors that influence the eradication of SCH japonica and support policymakers in designing more effective control strategies. One key limitation of this work is the assumption that treated individuals immediately return to the susceptible class, which reflects real-world conditions in communities situated near infected water sources. Nonetheless, this assumption may overestimate reinfection rates and consequently underestimate the long-term effectiveness of chemotherapy alone.

## Conclusions

This paper presents a mathematical model designed to investigate the intricate transmission dynamics of SCH japonica, a parasitic disease affecting both humans and animals. The model incorporates human factors and considers the involvement of animals, snails, and schistosome larvae. This inclusive approach allows us to delve into the intricate transmission complexities of the infection, shedding light on its zoonotic nature and transmission tendencies.

Examining the transmission dynamics concerning humans, our findings reveal that SCH spreads more rapidly in households near contaminated water sources than in households farther away. Similarly, animals with greater access to contaminated water are at a higher risk of infection than their counterparts with limited access. This insight into transmission patterns contributes to a comprehensive understanding of the disease’s dynamics across human and animal populations.

This paper aims to demonstrate and evaluate the impact of individual control strategies and the One Health approach in addressing SCH infection. Overall, the implementation of WaSH practices results in a greater reduction in human infection cases compared to the other interventions examined. Similarly, pasture prohibition leads to a more substantial decrease in animal infection cases than the other strategies. These findings suggest that interventions focused on interrupting transmission from snail to human and snail to animal achieve greater reductions in infection rates than chemotherapy-based approaches.

However, the persistent environmental reservoir of SCH japonica, even with reduced human infections through WaSH or diminished animal infections through pasture prohibition, underscores the need for a holistic One Health control strategy. Sustainable control programs can be formulated to achieve desired health outcomes by incorporating cost considerations and adopting a One Health approach that recognizes the interconnectedness between humans, animals, and their shared environment. The success of such programs relies on the full cooperation and commitment of stakeholders and relevant institutions [2].

Future work should build upon these findings by incorporating behavioral dynamics such as non-compliance, seasonal variation, and gradual adoption, and by validating the model with longer-term datasets. In addition, applying stochastic or probabilistic methods to quantify uncertainty and identifying Pareto-optimal strategies that balance effectiveness, feasibility, and cost would further strengthen policy relevance and support sustainable One Health interventions.

## Supplementary Information

Additional file 1.

## Data Availability

All relevant data used in the analyses are available within the manuscript and its Additional file 1 files. The model implementation, calibration scripts, datasets, and simulation codes have been deposited in a public GitHub repository and can be accessed at: https://github.com/NorvinBansilan/Schistosomiasis-japonica-.git. These resources may be used to reproduce the results presented in this study and to facilitate further model development.

## Abbreviations

SCH: Schistosomiasis
WaSH: Water access, sanitation, and hygiene
PRCC: Partial rank correlation coefficients
WHO: World Health Organization
LGU: Local Government Unit
DOH: Department of Health
MDA: Mass drug administration
SRFI: Swamps, rice fields, or irrigation areas
ZOOTRIP: Zoonotic transmission of intestinal parasites
LHS: Latin Hypercube sampling
