# Supplementary material for: Schistosomiasis japonica transmission dynamics: mathematical modeling in guiding One Health approach control strategies
Source: Infect Dis Poverty. 2026 Jan 9;15:5. doi: 10.1186/s40249-025-01404-7 (PMC12784478; doi:10.1186/s40249-025-01404-7)
Supplement: Supplementary file 1 — Additional file 1. [file 40249_2025_1404_MOESM1_ESM.pdf]

SUPPLEMENTARY INFORMATION

Schistosomiasis japonica transmission dynamics: mathematical modeling in guiding One Health approach control strategies

Norvin P. Bansilan[a,*], Joaquin M. Prada[b,*], Allen Jethro I. Alonte[c], Martha Elizabeth Betson[d], Vachel Gay V. Paller[c], Jomar F. Rabajante[a]

[a]*Institute of Mathematical Sciences, University of the Philippines Los Baños, Laguna, Philippines*
[b]*School of Veterinary Medicine, Faculty of Health and Medical Sciences, University of Surrey, Guildford, United Kingdom*
[c]*Institute of Biologucal Sciences, University of the Philippines Los Baños, Laguna, Philippines*
[d]*School of Veterinary Medicine, Department of Veterinary Epidemiology and Public Health, University of Surrey, Guildford, United Kingdom*

Abstract

Schistosomiasis (SCH) japonica remains a persistent public health concern in the Philippines despite continuing control efforts. This study aims to examine the transmission dynamics of SCH japonica and evaluate different intervention strategies using a One Health modeling approach, with the goal of supporting feasible control and elimination targets. We developed a compartmental mathematical model calibrated using field survey data collected in 2022 from eight endemic barangays in Agusan del Sur and Surigao del Norte. The dataset included SCH prevalence, egg excretion levels in humans and animals quantified through Kato-Katz, modified McMaster, and sedimentation techniques, and household distance to potential transmission sites. Multiple intervention strategies were examined, including human and animal chemotherapy, WaSH (water access, sanitation, and hygiene) adoption, pasture prohibition, vegetation clearing, and snail control. Sensitivity analysis using Partial Rank Correlation Coefficients (PRCC) was performed to identify influential transmission drivers. The model estimates baseline prevalence at approximately 20% in humans across the study areas. Under medium WaSH adoption, human prevalence is projected to decline to approximately 1.01% by 2030, whereas high WaSH coverage further reduces prevalence to 0.64%. Combining WaSH and pasture prohibition alongside chemotherapy is projected to reduce human prevalence to 0.094% and animal prevalence to 0.10% by 2030. Sensitivity analysis identified snail-to-human transmission rate (PRCC = 0.612) and snail shedding rate (PRCC = 0.607) as the most influential parameters. Integrated strategies focusing on WaSH, reduced animal exposure, and targeted chemotherapy offer the most effective pathway toward achieving World Health Organization's (WHO's) 2030 SCH targets. Implementation should be strengthened through health education, behavioral interventions, mechanization support, and active Local Government Unit participation.

Keywords: schistosomiasis japonica, mathematical modelling, one health, neglected tropical disease, macroparasite

Table of Contents

1. Life Cycle of *Schistosoma japonicum.* Diagram
2. Description of Model Variables
3. Mathematical Model
4. Epidemiological Data
5. Parameter Values
6. Model Interventions
7. Individual Interventions
8. One Health Approach
9. Sample Combination of Interventions
10. Sensitivity Analysis
11. Mathematical Analysis of the Model
12. References

*Corresponding author
Email addresses: npbansilan@up.edu.ph (Norvin P. Bansilan), j.prada@surrey.ac.uk (Joaquin M. Prada), aialonte@up.edu.ph (Allen Jethro I. Alonte), m.betson@surrey.ac.uk (Martha Elizabeth Betson), vvpaller@up.edu.ph (Vachel Gay V. Paller), jfrabajante@up.edu.ph (Jomar F. Rabajante)

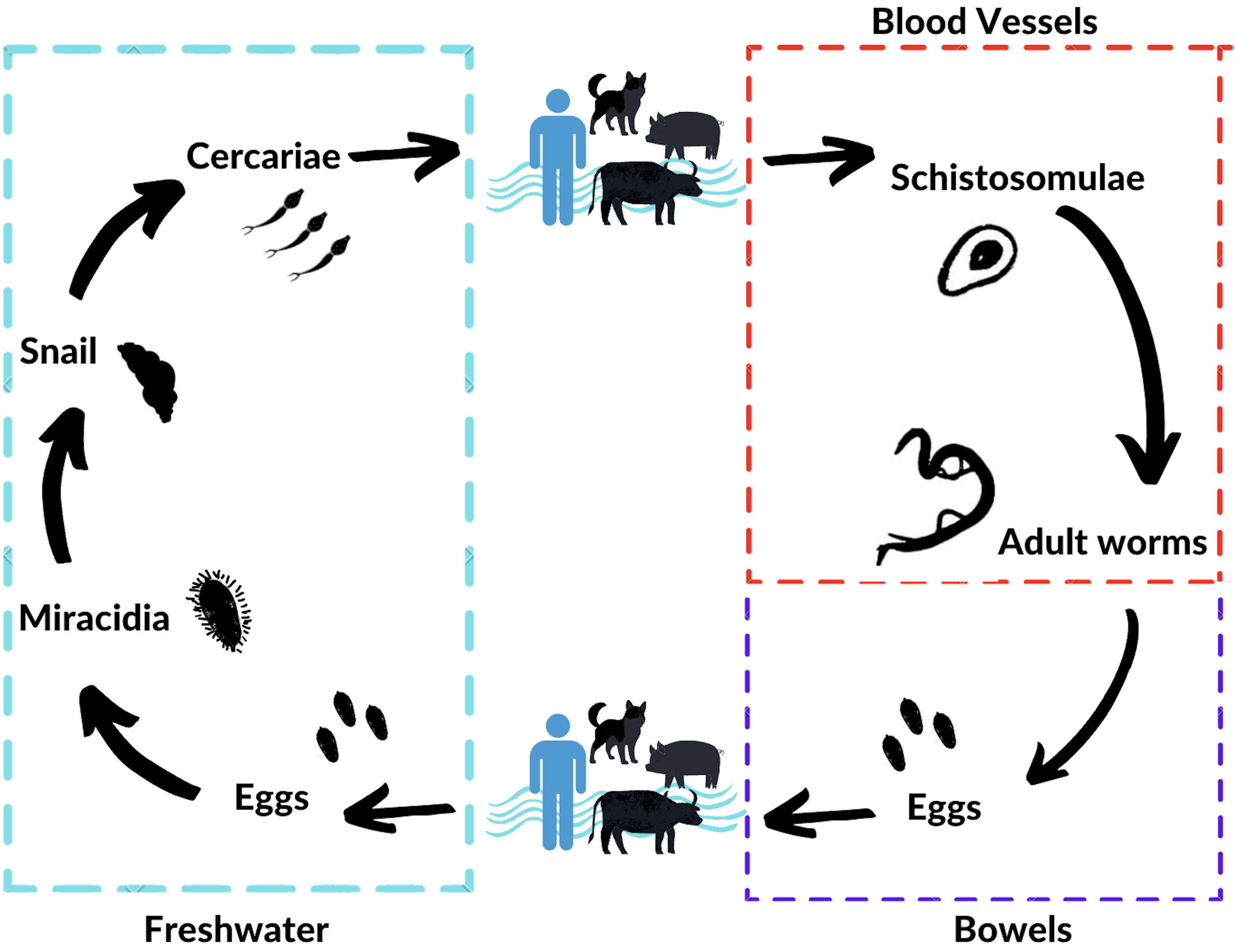

Figure S1: Life Cycle of *Schistosoma japonicum*. From the definitive host, eggs are expelled into freshwater. Subsequently, these eggs undergo hatching, giving rise to miracidia that infect a snail. Following a period, the snail releases cercariae, which then infects the definitive host, eventually transforming into schistosomula. These schistosomula navigate through the host's body, maturing into adult worms. Ultimately, adult schistosomes lay eggs, completing the cycle and initiating a new round of reproduction.

Table S1: Description of Variables for Schistosomiasis japonica Model. Initial Values are Based on the Field Data

Variable	Description	Values
S_{C_1}	Susceptible children far from the SRFI	12,286
S_{C_2}	Susceptible children near the SRFI	4027
S_{A_1}	Susceptible adults far from the SRFI	14,824
S_{A_2}	Susceptible adults near the SRFI	4,700
S_{F_1}	Susceptible farm animals	121
S_{F_2}	Susceptible companion animals	248
S_S	Susceptible snails	101
E_{C_1}	Exposed children far from the SRFI	0
E_{C_2}	Exposed children near the SRFI	0
E_{A_1}	Exposed adults far from the SRFI	0
E_{A_2}	Exposed adults near the SRFI	0
E_{F_1}	Exposed farm animals	0
E_{F_2}	Exposed companion animals	0
E_S	Exposed snails	0
I_{C_1}	Infected children far from the SRFI	267
I_{C_2}	Infected children near the SRFI	292
I_{A_1}	Infected adults far from the SRFI	633
I_{A_2}	Infected adults near the SRFI	73
I_{F_1}	Infected farm animals	3
I_{F_2}	Infected companion animals	6
I_S	Infected snails	6
M_1	Miracidia (from children to SRFI to snail)	30,087,650
M_2	Miracidia (from adults to SRFI to snail)	15,258,391
M_3	Miracidia (from animals to SRFI to snail)	111,402
Cer	Cercariae (from snail to SRFI to humans/animals)	5821
N_{H_1}	Total children population far from the SRFI	
N_{H_2}	Total children population near the SRFI	
N_{H_3}	Total adults population far from the SRFI	
N_{H_4}	Total adults population near the SRFI	
N_{Hf}	Total human population far from the SRFI	
N_{Hn}	Total human population near the SRFI	
N_{F_1}	Total farm animal population	
N_{F_2}	Total companion animal population	
N_S	Total snail population	

Mathematical Model for Children Far from the Swamp/Rice Fields/Irrigation

$$\frac{dS_{C_1}}{dt} = \overbrace{a_H N_{H_1}(1-\frac{N_{H_1}}{K_{H_1}})}^{S_{C_1}\ logistic\ growth} - \overbrace{JN_{Hf}}^{S_{C_1}\to S_{A_1}} + \overbrace{q_1 I_{C_1}}^{I_{C_1}\ recovery} - \overbrace{b_1\frac{Cer}{c}S_{C_1}}^{S_{C_1}\ interaction\ with\ Cer} - \overbrace{u_H S_{C_1}}^{S_{C_1}\ death}$$

$$\frac{dE_{C_1}}{dt} = \overbrace{b_1\frac{Cer}{c}S_{C_1}}^{S_{C_1}\ interaction\ with\ Cer} - \overbrace{l_h E_{C_1}}^{E_{C_1}\ latency} - \overbrace{u_H E_{C_1}}^{E_{C_1}\ death}$$

$$\frac{dI_{C_1}}{dt} = \overbrace{l_h E_{C_1}}^{E_{C_1}\ latency} - \overbrace{u_H I_{C_1}}^{I_{C_1}\ death} - \overbrace{q_1 I_{C_1}}^{I_{C_1}\ recovery}$$

Mathematical Model for Children Near the Swamp/Rice Fields/Irrigation

$$\frac{dS_{C_2}}{dt} = \overbrace{a_H N_{H_2}(1-\frac{N_{H_2}}{K_{H_2}})}^{S_{C_2}\ logistic\ growth} - \overbrace{JN_{Hn}}^{S_{C_2}\to S_{A_2}} + \overbrace{q_1 I_{C_2}}^{I_{C_2}\ recovery} - \overbrace{b_2\frac{Cer}{c}S_{C_2}}^{S_{C_2}\ interaction\ with\ Cer} - \overbrace{u_H S_{C_2}}^{S_{C_2}\ death}$$

$$\frac{dE_{C_2}}{dt} = \overbrace{b_2\frac{Cer}{c}S_{C_2}}^{S_{C_2}\ interaction\ with\ Cer} - \overbrace{l_h E_{C_2}}^{E_{C_2}\ latency} - \overbrace{u_H E_{C_2}}^{E_{C_2}\ death}$$

$$\frac{dI_{C_2}}{dt} = \overbrace{l_h E_{C_2}}^{E_{C_2}\ latency} - \overbrace{u_H I_{C_2}}^{I_{C_2}\ death} - \overbrace{q_1 I_{C_1}}^{I_{C_1}\ recovery}$$

Mathematical Model for Adults Far from the Swamp/Rice Fields/Irrigation

$$\frac{dS_{A_1}}{dt} = \overbrace{JN_{Hf}}^{S_{C_1}\to S_{A_1}} + \overbrace{q_1 I_{A_1}}^{I_{A_1}\ recovery} - \overbrace{b_3\frac{Cer}{c}S_{A_1}}^{S_{A_1}\ interaction\ with\ Cer} - \overbrace{u_H S_{A_1}}^{S_{A_1}\ death}$$

$$\frac{dE_{A_1}}{dt} = \overbrace{b_3\frac{Cer}{c}S_{A_1}}^{S_{A_1}\ interaction\ with\ Cer} - \overbrace{l_h E_{A_1}}^{E_{A_1}\ latency} - \overbrace{u_H E_{A_1}}^{E_{A_1}\ death}$$

$$\frac{dI_{A_1}}{dt} = \overbrace{l_h E_{A_1}}^{E_{A_1}\ latency} - \overbrace{u_H I_{A_1}}^{I_{A_1}\ death} - \overbrace{q_1 I_{A_1}}^{I_{A_1}\ recovery}$$

Mathematical Model for Adults Near the Swamp/Rice Fields/Irrigation

$$\frac{dS_{A_2}}{dt} = \overbrace{JN_{Hn}}^{S_{C_2}\to S_{A_2}} + \overbrace{q_1 I_{A_2}}^{I_{A_2}\ recovery} - \overbrace{b_4\frac{Cer}{c}S_{A_2}}^{S_{A_2}\ interaction\ with\ Cer} - \overbrace{u_H S_{A_2}}^{S_{A_2}\ death}$$

$$\frac{dE_{A_2}}{dt} = \overbrace{b_4\frac{Cer}{c}S_{A_2}}^{S_{A_2}\ interaction\ with\ Cer} - \overbrace{l_h E_{A_2}}^{E_{A_2}\ latency} - \overbrace{u_H E_{A_2}}^{E_{A_2}\ death}$$

$$\frac{dI_{A_2}}{dt} = \overbrace{l_h E_{A_2}}^{E_{A_2}\ latency} - \overbrace{u_H I_{A_2}}^{I_{A_2}\ death} - \overbrace{q_1 I_{A_2}}^{I_{A_2}\ recovery}$$

Mathematical Model for Farm Animals

$$\frac{dS_{F_1}}{dt} = \overbrace{a_{F_1} N_{F_1}(1-\frac{N_{F_1}}{K_{F_1}})}^{S_{F_1}\ logistic\ growth} + \overbrace{q_2 I_{F_1}}^{I_{F_1}\ recovery} - \overbrace{b_5\frac{Cer}{c}S_{F_1}}^{S_{F_1}\ interaction\ with\ Cer} - \overbrace{u_{F_1} S_{F_1}}^{S_{F_1}\ death}$$

$$\frac{dE_{F_1}}{dt} = \overbrace{b_5\frac{Cer}{c}S_{F_1}}^{S_{F_1}\ interaction\ with\ Cer} - \overbrace{l_{f_1} E_{F_1}}^{E_{F_1}\ latency} - \overbrace{u_{F_1} E_{F_1}}^{E_{F_1}\ death}$$

$$\frac{dI_{F_1}}{dt} = \overbrace{l_{f_1} E_{F_1}}^{E_{F_1}\ latency} - \overbrace{u_{F_1} I_{F_1}}^{I_{F_1}\ death} - \overbrace{q_2 I_{F_1}}^{I_{F_1}\ recovery}$$

Mathematical Model for Companion Animals

$$\frac{dS_{F_2}}{dt} = \overbrace{a_{F_2}N_{F_2}(1-\frac{N_{F_2}}{K_{F_2}})}^{S_{F_2}\ logistic\ growth} + \overbrace{q_2 I_{F_2}}^{I_{F_2}\ recovery} - \overbrace{b_6\frac{Cer}{c}S_{F_2}}^{S_{F_2}\ interaction\ with\ Cer} - \overbrace{u_{F_2}S_{F_2}}^{S_{F_2}\ death}$$

$$\frac{dE_{F_2}}{dt} = \overbrace{b_6\frac{Cer}{c}S_{F_2}}^{S_{F_2}\ interaction\ with\ Cer} - \overbrace{l_{f_2}E_{F_2}}^{E_{F_2}\ latency} - \overbrace{u_{F_2}E_{F_2}}^{E_{F_2}\ death}$$

$$\frac{dI_{F_2}}{dt} = \overbrace{l_{f_2}E_{F_2}}^{E_{F_2}\ latency} - \overbrace{u_{F_2}I_{F_2}}^{I_{F_2}\ death} - \overbrace{q_2 I_{F_2}}^{I_{F_2}\ recovery}$$

Mathematical Model for Snails

$$\frac{dS_S}{dt} = \overbrace{(f_s S_S + f_e E_S + f_i I_S)(1-\frac{N_S}{K_S})}^{S_S\ logistic\ growth} - \overbrace{i_1 S_S}^{S_S\ death} - \overbrace{e\frac{M_1}{c}S_S}^{S_S\ interaction\ with\ M_1} - \overbrace{e\frac{M_2}{c}S_S}^{S_S\ interaction\ with\ M_2} - \overbrace{e\frac{M_3}{c}S_S}^{S_S\ interaction\ with\ M_3}$$

$$\frac{dE_S}{dt} = \overbrace{e\frac{M_1}{c}S_S}^{S_S\ interaction\ with\ M_1} + \overbrace{e\frac{M_2}{c}S_S}^{S_S\ interaction\ with\ M_2} + \overbrace{e\frac{M_3}{c}S_S}^{S_S\ interaction\ with\ M_3} - \overbrace{i_2 E_S}^{E_S\ death} - \overbrace{yE_S}^{E_S\ prepatent}$$

$$\frac{dI_S}{dt} = \overbrace{yE_S}^{E_S\ prepatent} - \overbrace{i_2 I_S}^{I_S\ death}$$

Mathematical Model for Miracidia

$$\frac{dM_1}{dt} = \overbrace{(r_{C_1}I_{C_1} + r_{C_2}I_{C_2})g_1}^{eggs\ excreted\ by\ infected\ children} - \overbrace{d_m M_1}^{M_1\ death} - \overbrace{eN_S\frac{M_1}{c}}^{Snail\ interaction\ with\ M_1}$$

$$\frac{dM_2}{dt} = \overbrace{(r_{A_1}I_{A_1} + r_{A_2}I_{A_2})g_2}^{eggs\ excreted\ by\ infected\ adults} - \overbrace{d_m M_2}^{M_2\ death} - \overbrace{eN_S\frac{M_2}{c}}^{Snail\ interaction\ with\ M_2}$$

$$\frac{dM_3}{dt} = \overbrace{(r_{F_1}I_{F_1} + r_{F_2}I_{F_2})g_3}^{eggs\ excreted\ by\ infected\ animals} - \overbrace{d_m M_3}^{M_3\ death} - \overbrace{eN_S\frac{M_3}{c}}^{Snail\ interaction\ with\ M_3}$$

Mathematical Model for Cercariae

$$\frac{dCer}{dt} = \overbrace{sI_S}^{cercariae\ shed\ by\ infected\ snail} - \overbrace{d_c Cer}^{Cer\ death}$$

Table S2: Agusan del Sur, Philippines Epidemiological Data

Municipality	Bunawan	Trento	Bayugan	Esperanza
Barangay	Libertad	Manat	Taglatawan	Hawilian
Eggs Excreted by an Infected Children	222.9 epg	268.8 epg	0.0 epg	12.0 epg
Eggs Excreted by an Infected Adults	62.4 epg	0.0 epg	12.0 epg	24.0 epg
Eggs Excreted by an Infected Animals	20.7 epg	34.0 epg	0.0 epg	0.0 epg
Total Adult Population	79	63	87	92
Total Children Population	89	82	33	41
Total Animal Population	49	69	17	22
Total Snail Population	27	0	21	38
Average distance of snail sites to households	1.2 km	1.5 km	0.7 km	1.0 km

Table S3: Agusan del Sur, Philippines Epidemiological Data

Municipality	Mainit	San Isidro	Claver	Gigaquit
Barangay	San Isidro	Buhing Calipay/ Del Pilar	Daywan	San Isidro
Eggs Excreted by an Infected Children	24.0 epg	49.5 epg	24.0 epg	12.0 epg
Eggs Excreted by an Infected Adults	72.0 epg	68.0 epg	84.0 epg	108.0 epg
Eggs Excreted by an Infected Animals	27.5 epg	61.5 epg	0.0 epg	0.0 epg
Total Adult Population	59	91	86	63
Total Children Population	122	78	40	47
Total Animal Population	67	64	48	33
Total Snail Population	0	0	0	15
Average distance of snail sites to households	0.2 km	0.4 km	0.7 km	1.7 km

Parameter Values

The Philippines' current human birth rate in 2022 is 19.778 births per 1000 people [1]. So there are $\frac{19.778}{1000} = 0.019778$ births per person and $\frac{0.019778}{365.25} = 0.00005414921$ births every day. On the other hand, in general, the birth rate for companion animals (cat and dog) is 20% per year or 0.000547945206 per day, while the birth rate for farm animals (cow and carabao) is 10% per year or 0.000273972603 per day. Furthermore, from 2005 to 2010, the life expectancy for females born in the Philippines was 71.7 years [2]. The death rate is calculated as the inverse of life expectancy, which is $\frac{1}{71.7} = 0.013947001$ per year, or $\frac{0.013947001}{365} = 0.000038$ per day. On the other hand, the average life expectancy for companion animals and farm animals is 14 and 20 years, respectively. So, the death rate for companion animals is 0.000195694716 per day, while the death rate for farm animals is 0.000136986301 per day [3] [4] [5] [6] [7] [8] [9] [10] [11] [12] [13].

By multiplying the total number of each population (based on our data) by 100, the maximum number of each population is achieved. The proportion of children who become adults after a year is derived using data from the populations of Agusan del Sur and Surigao del Norte [14]. The proportion (in days) of the total population of 19 years old and the total population of all ages is calculated as $\frac{22{,}163}{1{,}182{,}069} = \frac{0.018749329}{365} = 0.000051368$. Furthermore, we used data from [15] to calculate the recovery rate among infected humans, which is 0.2 to 0.333 per year or 0.00054757 to 0.000911704 per day. Since our simulations assumed the disease's worst-case scenario, we set the recovery rate of 0.00055 among infected humans per day. Similarly, we used data from [15] to calculate the human latent period, which ranges from 25 to 35 days. Because our simulations considered the disease's worst-case spread, we chose a latent period of 25 days. We assumed that the parasite's lifespan in human and animal bodies is the same.

The transmission rates (from snail to human/animal) were manually estimated using the highest prevalence rate occur in Agusan del Sur and Surigao del Norte in the year 2008. We obtain the infection transmission rates from snails to children and adults near the SRFI at 0.00001227 per day. Also, assuming that farm animals transmit at the same rate as humans near the SRFI. However, the transmission rates of infection from snails to children, adults, and companion animals are set ten times lower than the previous transmission rates, i.e. 0.000001227 per day [16].

We calculate the water in the SRFI by assuming one snail in every 1,000 liters of water in the SRFI. Since we have 101 snails in our sample population, 101 × 1,000 = 101,000 liters. We set 100,000 liters of water in the SRFI.

In setting the parameter values for snail biology, we considered the disease's worst side spread. The fecundity of susceptible snails is 5.71/day, the fecundity of exposed and infected snails is 0.68/day, the mortality rate of uninfected snails is 0.001/day, the mortality rate of infected snails is 0.00886/day, exposure rate of snails to miracidia is 21.42857/day, the prepatent period in snails is 18 days and the shedding rate of snails is 971/day [17] [18] [16].

Fecalysis, also known as stool analysis, counts the number of eggs found in the feces of infected humans and animals in our sample population. Since we are considering the worst side spread of the disease, we take the most number of eggs excreted by the infected children, adults, and animals, i.e., 269 eggs per gram, 108 eggs per gram, and 62 eggs per gram, respectively. Humans excreted 200 grams of feces each day on average [19]. Thus, we get 269 × 200 = 53,800 eggs excreted per day, 108 × 200 = 21,600 eggs per day, and 62 × 200 = 12,400 eggs expelled per day. These expelled eggs eventually reach the water and hatch. The probability of eggs reaching water and hatching in each population is assumed. The eggs from children near the SRFI, adults near the SRFI, and farm animals are assigned a probability of 0.99 of reaching water and hatching. While the eggs from children far the SRFI, adults far the SRFI, and companion animals are set to 0.495, which is half the probability of the former populations.

Furthermore, miracidia have a lifespan of 4 to 16 hours or 0.166667 to 0.666667 days, whereas cercariae have a lifespan of 8 to 20 hours or 0.333333 to 0.833333 days [20]. We determine miracidia and cercariae mortality rates as the inverse of the lifespan. Again, we considered the disease's worst negative effects. As a result, we choose the parasites with the longest lifespans, 0.666667 days for miracidia and 0.833333 days for cercariae, respectively. As a result, $\frac{1}{0.666667} = 1.5$ per day and $\frac{1}{0.833333} = 1.2$ per day.

The initial conditions for our system of ordinary differential equations are
$S_{C_1}(0) \geq 0$, $E_{C_1}(0) \geq 0$, $I_{C_1}(0) \geq 0$, $S_{C_2}(0) \geq 0$, $E_{C_2}(0) \geq 0$, $I_{C_2}(0) \geq 0$,
$S_{A_1}(0) \geq 0$, $E_{A_1}(0) \geq 0$, $I_{A_1}(0) \geq 0$, $S_{A_2}(0) \geq 0$, $E_{A_2}(0) \geq 0$, $I_{A_2}(0) \geq 0$,
$S_{F_1}(0) \geq 0$, $E_{F_1}(0) \geq 0$, $I_{F_1}(0) \geq 0$, $S_{F_2}(0) \geq 0$, $E_{F_2}(0) \geq 0$, $I_{F_2}(0) \geq 0$,
$S_S(0) \geq 0$, $E_S(0) \geq 0$, $I_S(0) \geq 0$, $M_1(0) \geq 0$, $M_2(0) \geq 0$,$M_3(0) \geq 0$, and $Cer(0) \geq 0$.

Table S4: Parameters of the Schistosomiasis japonica Model. Calculated and assumed values are based on the biological and ecological characteristics of human and animals. For uniformity, we used days as units.

Parameter	Description	Values	References
a_H	Birth rate of human	0.00005414921/day	[1]
a_{F_1}	Birth rate of farm animals	0.000273972603/day	[3] [4] [7] [8] [9] [13]
a_{F_2}	Birth rate of companion animals	0.000547945206/day	[5] [6] [10] [11] [12]
u_H	Death rate of human	0.000038/day	Estimated
u_{F_1}	Death rate of farm animals	0.000136986301/day	[3] [4] [7] [8] [9] [13]
u_{F_2}	Death rate of companion animals	0.000195694716/day	[5] [6] [10] [11] [12]
K_{H_1}	Maximum number of children far from the SRFI	1,228,600	Estimated
K_{H_2}	Maximum number of children near the SRFI	402,700	Estimated
K_{H_3}	Maximum number of adults far from the SRFI	1,482,400	Estimated
K_{H_4}	Maximum number of adults near the SRFI	470,000	Estimated
K_{F_1}	Maximum number of farm animals	12,100	Estimated
K_{F_2}	Maximum number of companion animals	24,800	Estimated
K_S	Maximum number of snails in the environment	10,100	Estimated
J	Proportion of children mature into adults after a year	0.000051368	Estimated
q_1	Recovery rate among infected humans	0.00055/day	Estimated
q_2	Recovery rate among infected animals	0.00055/day	Estimated
b_1	Transmission rate of snail-to-children far from the SRFI	0.000001227/day	Estimated
b_2	Transmission rate of snail-to-children near the SRFI	0.00001227/day	Estimated
b_3	Transmission rate of snail-to-adults far from the SRFI	0.000001227/day	Estimated
b_4	Transmission rate of snail-to-adults near the SRFI	0.00001227/day	Estimated
b_5	Transmission rate of snail-to-farm animals	0.00001227/day	Estimated
b_6	Transmission rate of snail-to-companion animals	0.000001227/day	Estimated
c	Water in the SRFI	100,000 liters	Estimated
$\frac{1}{l_h}$	Latent period in humans	25 days	Estimated
$\frac{1}{l_{f_1}}$	Latent period in farm animals	25 days	Estimated
$\frac{1}{l_{f_2}}$	Latent period in companion animals	25 days	Estimated
f_s	Fecundity of susceptible snails	5.71/day	[16]
f_e	Fecundity of exposed snails	0.68/day	[16]
f_i	Fecundity of infected snails	0.68/day	[16]
i_1	Mortality rate of uninfected snails	0.001/day	[18]
i_2	Mortality rate of infected snails	0.00886/day	[18]
e	Exposure rate of snails to miracidia	21.42857/day	[16]
$\frac{1}{y}$	Prepatent period in snails	18 days	[15]
s	Shedding rate of snails	971/day	[17]
r_{C_1}	Probability of eggs reaching water and hatching (children far from the SRFI)	0.495	Estimated
r_{C_2}	Probability of eggs reaching water and hatching (children near the SRFI)	0.99	Estimated
r_{A_1}	Probability of eggs reaching water and hatching (adults far from the SRFI)	0.495	Estimated
r_{A_2}	Probability of eggs reaching water and hatching (adults near the SRFI)	0.99	Estimated
r_{F_1}	Probability of eggs reaching water and hatching (farm animals)	0.99	Estimated
r_{F_2}	Probability of eggs reaching water and hatching (companion animals)	0.495	Estimated
g_1	Eggs excreted by an infected children	53,800/day	Data
g_2	Eggs excreted by an infected adults	21,600/day	Data
g_3	Eggs excreted by an infected animals	12,400/day	Data
d_m	Death rate of miracidia	1.5/day	[20]
d_c	Death rate of cercariae	1.2/day	[20]

Model Interventions

Human chemotherapy directly affects the parameter q_1, which is the recovery rate among infected humans, while animal chemotherapy directly affects the parameter q_2, which is the recovery rate among infected animals [15]. On the other hand, WaSH has a direct effect on the parameters b_1, b_2, b_3, and b_4, which represent the snail-to-human transmission rates [16]. The WaSH also affects the parameters on the probability of eggs reaching water and hatching on humans (r_{C_1}, r_{C_2}, r_{A_1}, and r_{A_2}). In the same manner, pasture prohibition directly affects the parameters b_5 and b_6, which represent snail transmission rates to animals. Moreover, snail control directly impacts the parameters i_1 and i_2, which represent snail mortality.

Indeed, we set the currently used values of the model parameters if there is no implementation of an intervention. We set $q_1 = 0.00055$ among infected humans per day in human chemotherapy without implementation of an intervention. However, we choose the other value of $q_1 = 0.0009$ among infected humans per day with medium implementation of human chemotherapy. Using the proportion of the values of without and medium implementation, we derived the value of high human chemotherapy. Thus, if $\frac{00055}{0.0009} = 0.601$ then $\frac{0.0009}{0.601} = 0.00152$. We now set the value of high human chemotherapy to $q_1 = 0.00152$ among infected humans per day. Similarly, if we assume that the recovery rate among infected humans and infected animals is the same, we get $q_2 = 0.00055$/day for without, $q_2 = 0.0009$/day for medium, and $q_2 = 0.00152$/day for high implementation of an intervention.

In WaSH, we set $b_2 = b_4 = 0.00001227$/day if without intervention is implemented. Now, with medium WaSH, we decrease the value by a magnitude of 100, thus, $b_2 = b_4 = 0.0000001227$/day. For the value of high implementation of WaSH, we again decrease the value of medium WaSH by a magnitude of 100, hence we set $b_2 = b_4 = 0.000000001227$/day. Because b_2 and b_4 are assumed to have the same value with b_5, as a result, $b_5 = 0.00001227$/day without pasture prohibition, $b_5 = 0.0000001227$/day for medium pasture prohibition and $b_5 = 0.000000001227$/day for high pasture prohibition. On the other hand, we set $b_1 = b_3 = 0.000001227$/day if without intervention is implemented. Now, with medium WaSH, we decrease the value by a magnitude of 100, thus, $b_1 = b_3 = 0.00000001227$/day. For the value of the high implementation of WaSH, we again decrease the value of medium WaSH by a magnitude of 100, hence we set $b_1 = b_3 = 0.0000000001227$/day. Because b_1 and b_3 are assumed to have the same value with b_6, as a result, $b_6 = 0.000001227$/day without pasture prohibition, $b_6 = 0.00000001227$/day for medium pasture prohibition and $b_6 = 0.0000000001227$/day for high pasture prohibition.

Moreover, in WaSH, we set $r_{C_1} = r_{A_1} = 0.495$ and $r_{C_2} = r_{A_2} = 0.99$ if without intervention is implemented. Now, with medium WaSH, we divide the value by half so that $r_{C_1} = r_{A_1} = 0.2475$ and $r_{C_2} = r_{A_2} = 0.495$. For the value of high implementation of WaSH, we again divide the value of medium WaSH by half so that $r_{C_1} = r_{A_1} = 0.12375$ and $r_{C_2} = r_{A_2} = 0.2475$.

Snail control by vegetation clearing is considered a medium implementation, and snail control by vegetation clearing with molluscicides is considered a high implementation. The mortality rate of uninfected snails ranges from 0.001 to 0.022 per day. We set $i_1 = 0.001$/day in the absence of snail control. In the case of vegetation clearing, we choose the other value, $i_1 = 0.022$/day. We calculated the value of vegetation clearing with molluscicides by dividing the value of without snail control by the value of vegetation clearing. Thus, if $\frac{0.001}{0.022} = 0.0461$, then $\frac{0.022}{0.0461} = 0.472$. As a result, we set $i_1 = 0.472$/day. Furthermore, the mortality rate of infected snails ranges from 0.00886/day to 0.08671/day. We set $i_2 = 0.00886$/day in the absence of snail control. In the case of vegetation clearing, we choose the other value, $i_2 = 0.08671$/day. We used the proportion of these values without snail control and vegetation clearing, to get the value of vegetation clearing with molluscicides.. Thus, if $\frac{0.00886}{0.08671} = 0.1021$, then $\frac{0.08671}{0.1021} = 0.84896$. Hence, we set $i_2 = 0.84896$/day.

Table S5: Sample Combinations of Interventions

**Interventions**	**Combination 1**	**Combination 2**	**Combination 3**
Human chemotherapy	Without	Without	High
Animal chemotherapy	Medium	High	Without
WaSH (Water, Sanitation, and Hygiene)	High	Without	Medium
Pasture prohibition	Medium	High	Without
Snail control	Without	Vegetation clearing	Without

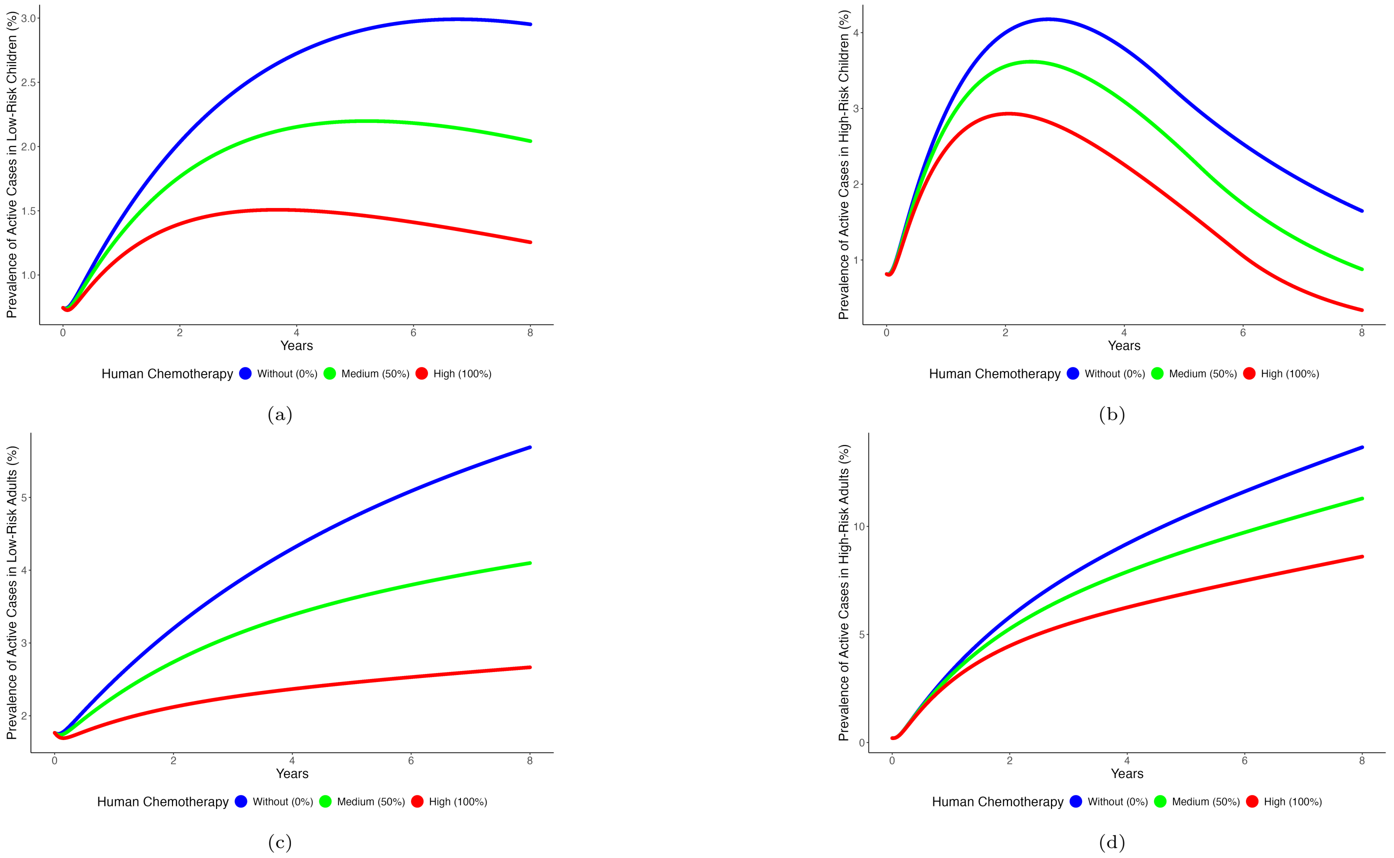

Figure S2: Prevalence of Active Cases in Infected Human Populations with Human Chemotherapy Implementation. The x-axes denote the eight-year implementation of human chemotherapy - Without (blue), Medium (green), and High (red). While y-axes denote the prevalence (in percent) of active cases in low-risk and high-risk populations.

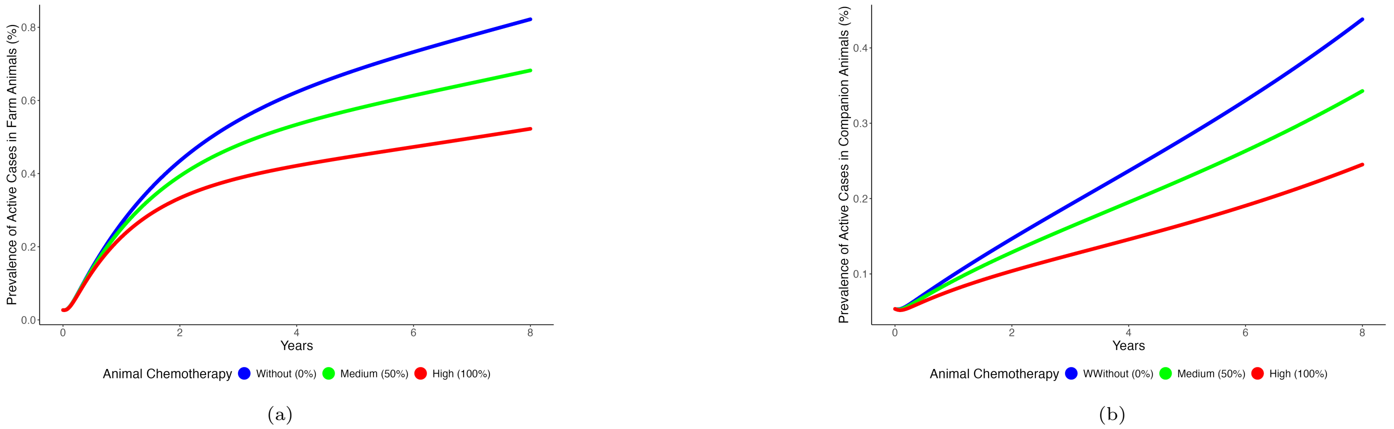

Figure S3: Prevalence of Active Cases in Infected Animal Populations with Animal Chemotherapy Implementation. The x-axes denote the eight-year implementation of animal chemotherapy - Without (blue), Medium (green), and High (red). While y-axes denote the prevalence (in percent) of active cases in farm and companion animal populations.

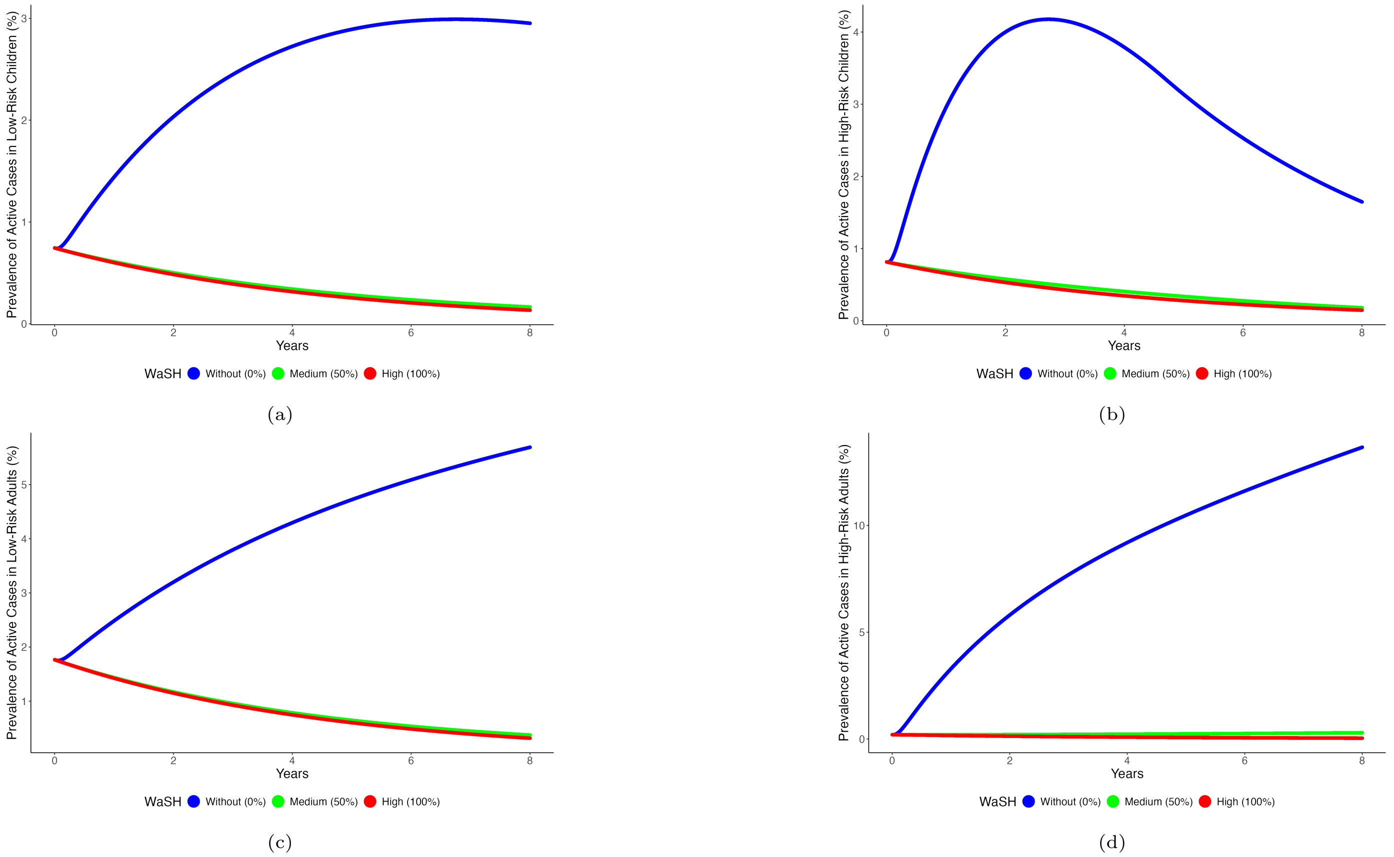

Figure S4: Prevalence of Active Cases in Infected Human Populations with WaSH Implementaion. The x-axes denote the eight-year implementation of WaSH - Without (blue), Medium (green), and High (red). While y-axes denote the prevalence (in percent) of active cases in low-risk and high-risk populations.

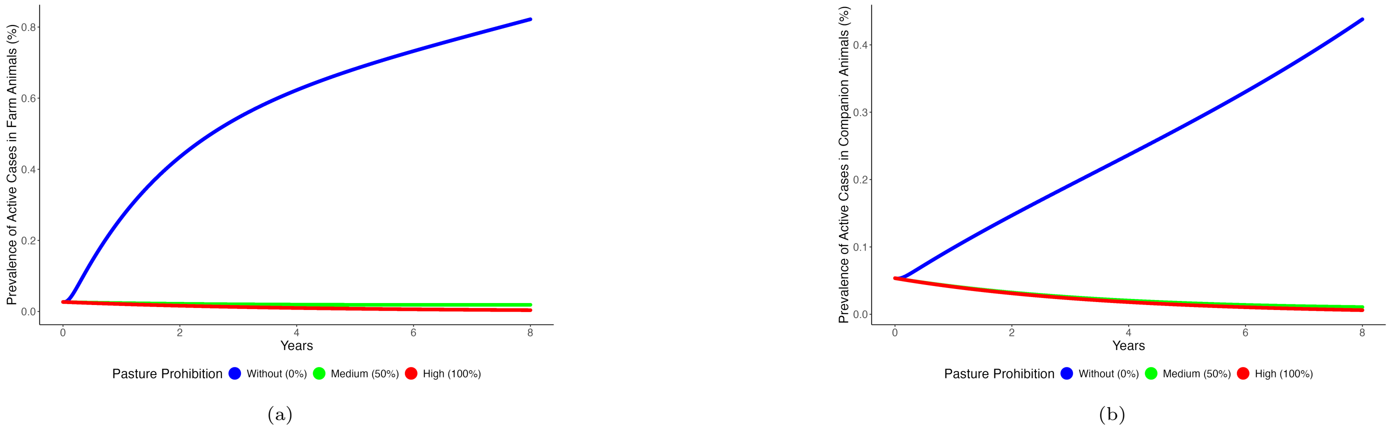

Figure S5: Prevalence of Active Cases in Infected Animal Populations with Pasture Prohibition Implementation. The x-axes denote the eight-year implementation of pasture prohibition - Without (blue), Medium (green), and High (red). While y-axes denote the prevalence (in percent) of active cases in farm and companion animal populations.

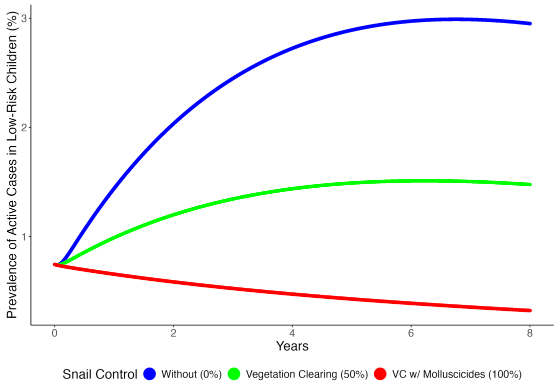

(a)

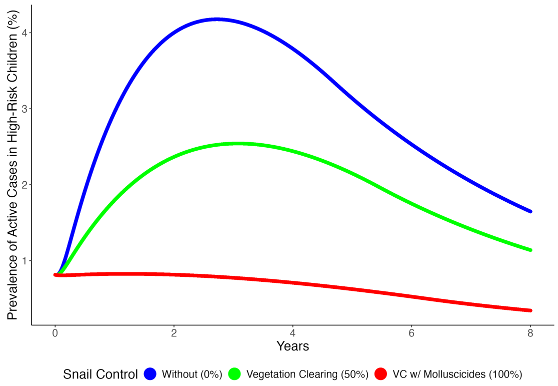

(b)

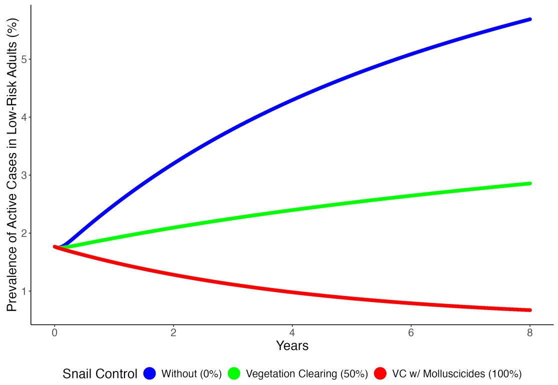

(c)

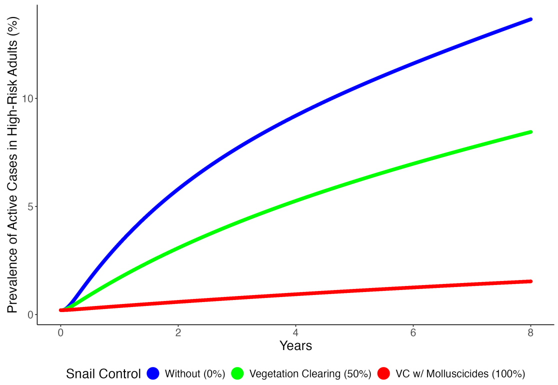

(d)

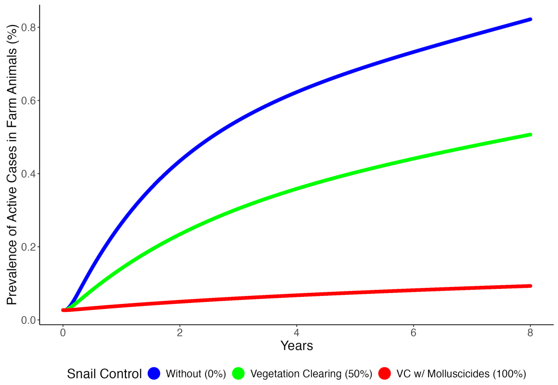

(e)

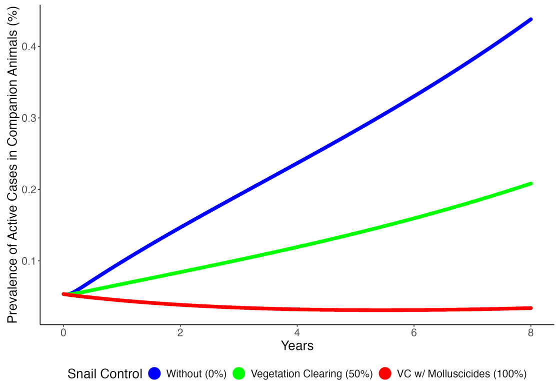

(f)

Figure S6: Prevalence of Active Cases in Infected Human and Animal Populations with Snail Control Implementation. The x-axes denote the eight-year implementation of snail control - Without (blue), Medium (green) and High (red). While y-axes denote the prevalence (in percent) of active cases in low-risk and high-risk populations of both humans and animals.

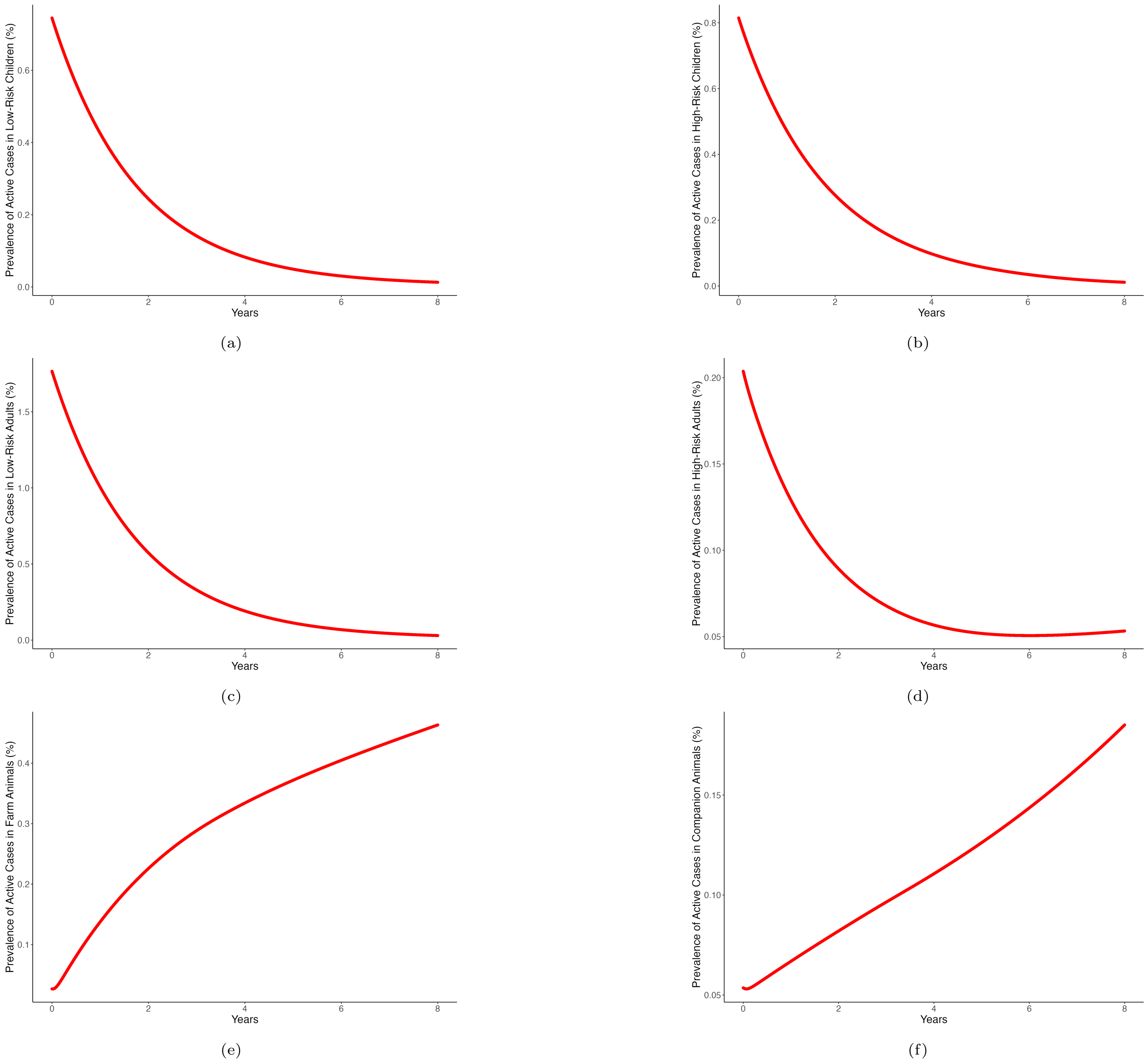

Figure S7: One Health Approach: High Human Chemotherapy, Medium WaSH and Vegetation Clearing. The x-axes denote the eight-year implementation of the One Health approach. While y-axes denote the prevalence (in percent) of active cases in low-risk and high-risk populations of both humans and animals.

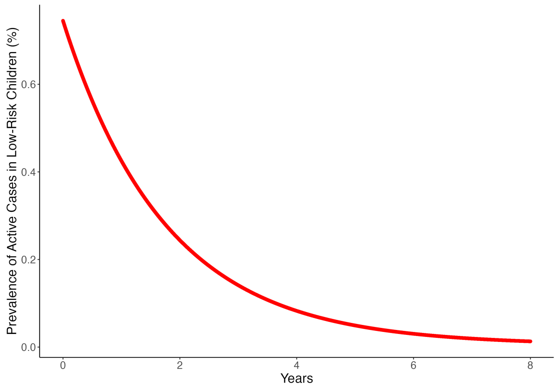

(a)

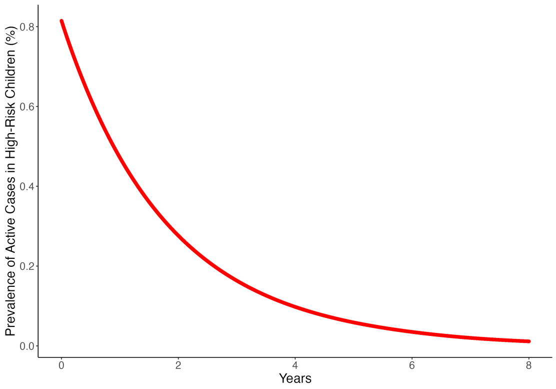

(b)

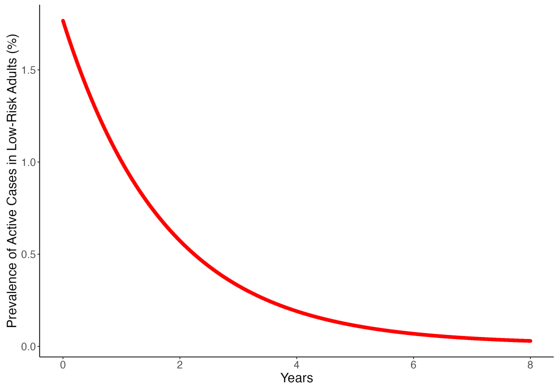

(c)

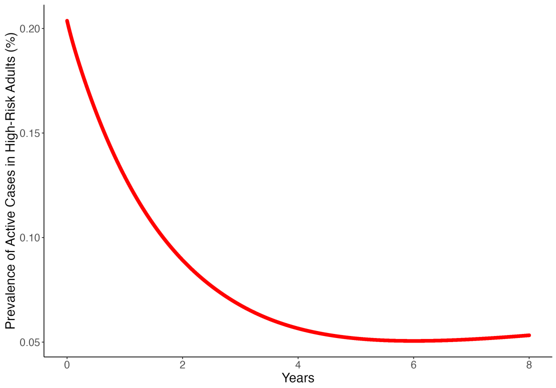

(d)

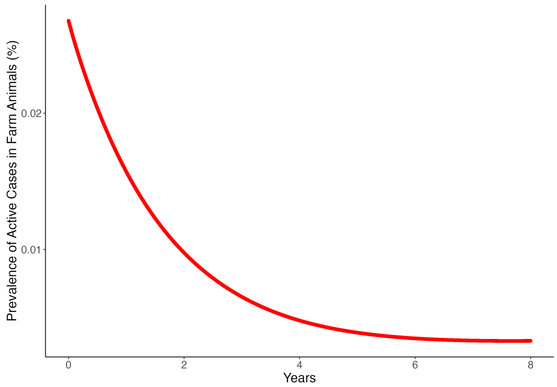

(e)

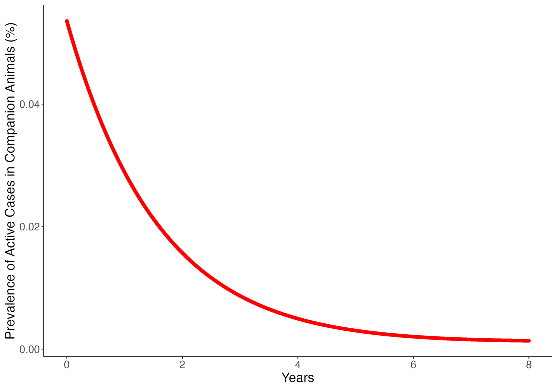

(f)

Figure S8: One Health Approach: Combinations of Interventions - High Human and Animal Chemotherapy, Medium WaSH and Pasture Prohibition, and Vegetation Clearing. The x-axes denote the eight-year implementation of the One Health approach. While y-axes denote the prevalence (in percent) of active cases in low-risk and high-risk populations of both humans and animals.

Table S6: Combination of Medium WaSH and High Human Chemotherapy

Province	Municipality	Barangay	Prevalence Rate (%)		
			**2022** (Field data)	**2026** (Predicted)	**2030** (Predicted)
Agusan del Sur	Bunawan	Libertad	3.27	2.72	0.98
	Trento	Manat	6.11	5.09	1.83
	Bayugan City	Taglatawan	1.77	1.48	0.53
	Esperanza	Hawilian	8.15	6.79	2.45
Surigao del Norte	Mainit	San Isidro	12.37	10.30	3.71
	San Isidro	Buhing Calipay/Del Pilar	21.27	17.71	6.38
	Claver	Daywan	7.35	6.12	2.21
	Gigaquit	San Isidro	5.56	4.63	1.67

Sensitivity Analysis

The sensitivity analysis using Partial Rank Correlation Coefficients (PRCC) identified the parameters with the greatest influence on the model outcomes across human, animal, and environmental domains (Figures X–Y).

For human-related parameters, the snail-to-human transmission rate (b_H, PRCC = 0.612) had the strongest positive association with prevalence, underscoring that increases in exposure to cercariae drive higher infection levels. The human birth rate (α_H, PRCC = 0.206) also showed a moderate positive effect, reflecting how population growth sustains the pool of susceptibles. In contrast, the volume of water in the environment (c, PRCC = –0.590) was negatively correlated, suggesting that greater water availability may dilute cercarial concentration and reduce transmission risk. Similarly, the human recovery rate (q_1, PRCC = –0.493) reduced prevalence, consistent with treatment or natural recovery effects.

For animal-related parameters, the carrying capacity of snails (K_s, PRCC = 0.604) emerged as the dominant positive driver, emphasizing the role of snail abundance in maintaining transmission. The prepatent period of snails (y, PRCC = 0.192) also contributed positively, while the mortality of infected snails (i_2, PRCC = –0.133) reduced prevalence. Other animal-related parameters had minimal influence.

Within the environmental domain, the snail shedding rate (s, PRCC = 0.607) was strongly and positively correlated with infection outcomes, reflecting the importance of cercarial release into water bodies. Conversely, cercarial death rate (d_c, PRCC = –0.593) had a strong negative correlation, highlighting the role of environmental conditions and interventions (e.g., vegetation clearing or molluscicides) that increase cercarial mortality in reducing transmission. Other environmental parameters exerted little influence.

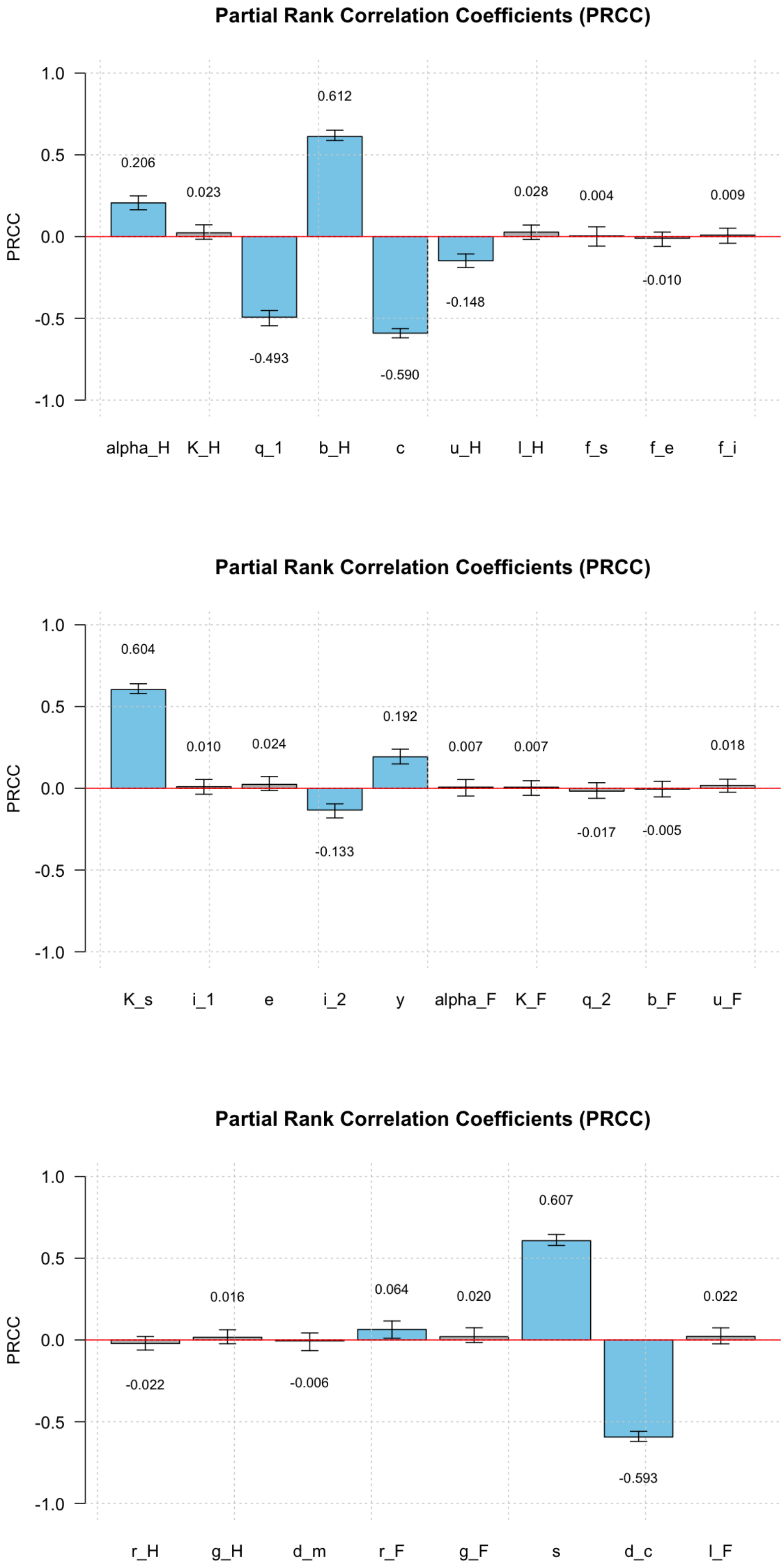

Figure S9: Sensitivity Analysis: Results of the sensitivity analysis based on 2,000 simulation runs using Latin Hypercube Sampling (LHS) and Partial Rank Correlation Coefficients (PRCC).

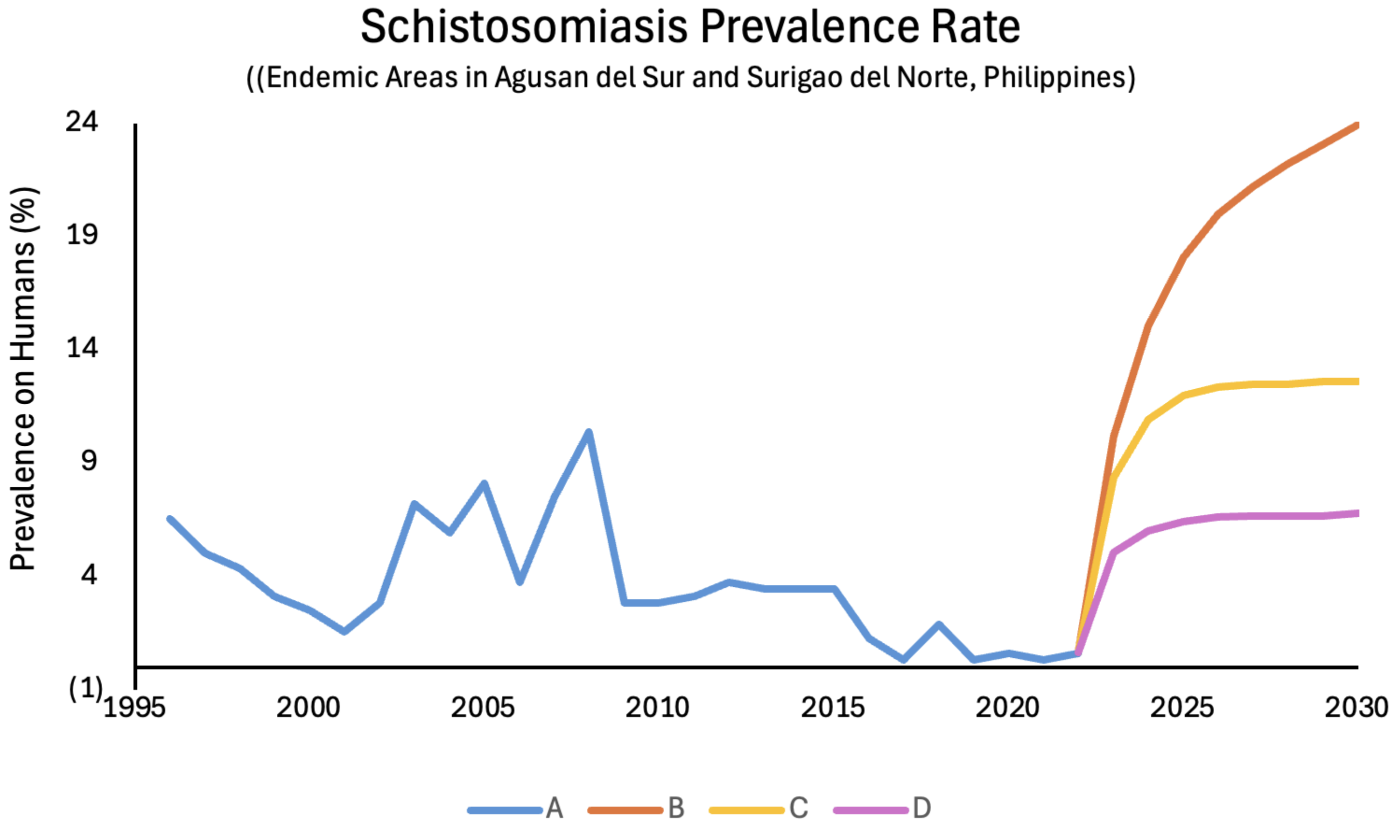

Figure S10: Schistosomiasis Prevalence Rate (in percent) on Humans in the endemic areas of Agusan del Sur and Surigao del Norte, Philippines from 1996 to 2030. The diagram shows the historical data of prevalence from 1996 to 2022 and simulated prevalence after eight years with different scenarios: A - historical data; B - without any intervention, C - with both high human and animal chemotherapy only; and D - with both high human and animal chemotherapy and vegetation clearing.

Mathematical Analysis of the Model

Steady-State Solutions

We consider a general equation to represent the human-definitive population and animal-reservoir population. Susceptible, exposed, and infected populations are denoted as S, E, and I, respectively. The total population of the host is given by $N = S + E + I$ and the maximum number population is assumed to be equal to K. The population birth rate is set to a constant a while the recovery rate is set to a constant q. Moreover, the transmission rate from snail to human/animal is denoted as b, the latent period is set to be l, and a death rate of u. Furthermore, miracidia M and cercariae Cer are assumed as a constant parameter i.e. larvae micaridia and cercariae are always in the environment.

$$\begin{cases} \frac{dS}{dt} = aN(1-\frac{N}{K}) + qI - b\frac{Cer}{c}S - uS \\ \frac{dE}{dt} = b\frac{Cer}{c}S - lE - uE \\ \frac{dI}{dt} = lE - uI - qI \\ \frac{dS_s}{dt} = (f_sS_s + f_eE_s + f_iI_s)(1-\frac{N_s}{K_s}) - i_1S_s - e\frac{M}{c}S_s \\ \frac{dE_s}{dt} = e\frac{M}{c}S_s - i_2E_s - yE_s \\ \frac{dI_s}{dt} = yE_s - i_2I_s \end{cases} \tag{1}$$

System (1) always has two steady-state solutions:

1. Disease Free Equilibrium (DFE) i.e. the infected populations are zero ($E = I = E_s = I_s = 0$), then (1) could be transformed into

$$\begin{cases} \frac{dS}{dt} = aS\left(1-\frac{S}{K}\right) - b\frac{Cer}{c}S - uS \\ \frac{dS_s}{dt} = f_sS_s(1-\frac{S_s}{K_s}) - i_1S_s - e\frac{M}{c}S_s \end{cases} \tag{2}$$

thus, we have

$$S = S_s = 0, \tag{3}$$

$$S = \frac{K(ac - bCer - uc)}{ac}, \tag{4}$$

and

$$S_s = \frac{K_s(f_sc - i_1c - eM)}{f_sc} \tag{5}$$

If $S = S_s = 0$, then

$$\text{DFE} = (0, 0, 0, 0, 0, 0)\,. \tag{6}$$

Otherwise,

$$\text{DFE} = \left(\frac{K(ac - bCer - uc)}{ac}, 0, 0, \frac{K_s(f_sc - i_1c - eM)}{f_sc}, 0, 0\right) \tag{7}$$

2. Endemic Equilibrium (EE) i.e. the infection is constantly maintained in the population, we have

$$\text{EE} = (S^*, E^*, I^*, S_s^*, E_s^*, I_s^*) \tag{8}$$

where

$$S^* = \frac{\frac{abCerK}{c(l+u)}+\frac{albCerK}{c(l+u)(u+q)}+aK+\frac{qlbCerK}{c(l+u)(u+q)}-\frac{bCerK}{c}-uK}{a+a(\frac{bCer}{c(l+u)})^2+a(\frac{lbCer}{c(l+u)(u+q)})^2+2a(\frac{bCer}{c(l+u)}+\frac{lbCer}{c(l+u)(u+q)}+\frac{l(bCer)^2}{(c(l+u))^2(u+q)})},$$

$$E^* = \frac{bCer\frac{\frac{abCerK}{c(l+u)}+\frac{albCerK}{c(l+u)(u+q)}+aK+\frac{qlbCerK}{c(l+u)(u+q)}-\frac{bCerK}{c}-uK}{a+a(\frac{bCer}{c(l+u)})^2+a(\frac{lbCer}{c(l+u)(u+q)})^2+2a(\frac{bCer}{c(l+u)}+\frac{lbCer}{c(l+u)(u+q)}+\frac{l(bCer)^2}{(c(l+u))^2(u+q)})}}{c(l+u)},$$

$$I^* = \frac{lbCer\frac{\frac{abCerK}{c(l+u)}+\frac{albCerK}{c(l+u)(u+q)}+aK+\frac{qlbCerK}{c(l+u)(u+q)}-\frac{bCerK}{c}-uK}{a+a(\frac{bCer}{c(l+u)})^2+a(\frac{lbCer}{c(l+u)(u+q)})^2+2a(\frac{bCer}{c(l+u)}+\frac{lbCer}{c(l+u)(u+q)}+\frac{l(bCer)^2}{(c(l+u))^2(u+q)})}}{c(l+u)(u+q)},$$

$$S_s^* = \frac{f_sK_s-i_1K_s-\frac{eMK_s}{c}+\frac{f_eeMK_s}{c(i_2+y)}+\frac{f_iyeMK_s}{c(i_2+y)(i_2)}}{f_s+f_e(\frac{eM}{c(i_2+y)})^2+f_i(\frac{yeM}{c(i_2+y)(i_2)})^2+\frac{(f_s+f_e)eM}{c(i_2+y)}+\frac{(f_s+f_i)yeM}{c(i_2+y)(i_2)}+\frac{(f_e+f_i)y(eM)^2}{(c(i_2+y))^2(i_2)}},$$

$$E_s^* = \frac{eM}{c(i_2+y)}\frac{f_sK_s-i_1K_s-\frac{eMK_s}{c}+\frac{f_eeMK_s}{c(i_2+y)}+\frac{f_iyeMK_s}{c(i_2+y)(i_2)}}{f_s+f_e(\frac{eM}{c(i_2+y)})^2+f_i(\frac{yeM}{c(i_2+y)(i_2)})^2+\frac{(f_s+f_e)eM}{c(i_2+y)}+\frac{(f_s+f_i)yeM}{c(i_2+y)(i_2)}+\frac{(f_e+f_i)y(eM)^2}{(c(i_2+y))^2(i_2)}},$$

and

$$I_s^* = \frac{yeM}{c(i_2+y)(i_2)}\frac{f_sK_s-i_1K_s-\frac{eMK_s}{c}+\frac{f_eeMK_s}{c(i_2+y)}+\frac{f_iyeMK_s}{c(i_2+y)(i_2)}}{f_s+f_e(\frac{eM}{c(i_2+y)})^2+f_i(\frac{yeM}{c(i_2+y)(i_2)})^2+\frac{(f_s+f_e)eM}{c(i_2+y)}+\frac{(f_s+f_i)yeM}{c(i_2+y)(i_2)}+\frac{(f_e+f_i)y(eM)^2}{(c(i_2+y))^2(i_2)}}. \tag{9}$$

Basic Reproduction Number

The basic reproduction number, R_0, is a threshold parameter to determine whether a disease will invade a population. In theory, if $R_0 < 1$, each infected person generates less than one new infected individual during their whole infectious period, implying that the infection will not persist in the population. If, on the other hand, $R_0 > 1$, each infected person infects more than one person, meaning that the disease will eventually invade the population. Using the next-generation matrix method, which Diekmann et al. [21] developed, R_0 of the model system (1) is given by

$$R_0 = \sqrt{(\frac{bCerK(ac-bCer-uc)y}{ac^2((i_2+y)(i_2))})(\frac{eMK_s(f_sc-i_1c-eM)l}{f_sc^2(l+u)(u+q)})}. \tag{10}$$

Looking at equation (10), R_0 increases if the values in the numerator increase. For example, if the transmission rate of infection from snail to human/animal b increases then R_0 increases. On the other hand, R_0 decreases if the values in the denominator increase. For example, if the mortality rate of the infected snails i_2 increases due to vegetation clearing with molluscicides then R_0 decreases.

Moreover, the disease-free steady state DFE is locally asymptotically stable when $R_0 < 1$ and unstable when $R_0 > 1$. The endemic equilibrium EE is locally asymptotically stable when $R_0 > 1$.

References

[1] U. N. D. of Economic, S. A. P. Dynamics, World population prospects 2019 (n.d.).
URL https://population.un.org/wpp/DataSources/608

[2] P. S. Authority, Life expectancy at birth of women (n.d.).
URL https://psa.gov.ph/content/life-expectancy-birth-women

[3] J. E. B. Riñon, R. Mendoza, A. A. de los Reyes V, V. Y. B. Jr, V. P. M. Mendoza, Management and control of schistosomiasis in agusan del sur, philippines: A modeling study, . (2022).

[4] P. S. Authority, Cattle situation report (n.d.).
URL https://psa.gov.ph/livestock-poultry-iprs/cattle/inventory

[5] dog breed information, Aspin (n.d.).
URL https://www.dogbreedinfo.com/a/aspin.htm

[6] C. B. FAQ, Puspin cat (pusang pinoy) breed complete information (n.d.).
URL https://catbreedsfaq.com/puspin-cat?expand_article=1

[7] A. De Vries, M. Marcondes, Overview of factors affecting productive lifespan of dairy cows, Animal 14 (S1) (2020) s155–s164.

[8] R. FARM, Carabao buffalo: Characteristics, origin and uses (n.d.).
URL https://www.roysfarm.com/carabao-buffalo/

[9] D. of Agriculture, Philippine livestock industry report (n.d.).
URL https://www.roysfarm.com/carabao-buffalo/

[10] W. A. Protection, Philippines: Animal welfare (n.d.).
URL https://paws.org.ph/

[11] P. A. W. Society, Stray cat population in the philippines (n.d.).
URL https://paws.org.ph/

[12] D. of Science, Technology, Pet ownership in the philippines (n.d.).
URL https://www.dost.gov.ph/

[13] T. P. D. of Agriculture, Cattle production in the philippines (n.d.).
URL https://documents1.worldbank.org/curated/en/781401468095352238/text/multi-page.txt

[14] P. S. Authority, 2020 census of population and housing (2020 cph) population counts declared official by the president (n.d.).
URL https://psa.gov.ph/content/2020-census-population-and-housing

[15] R. M. Anderson, R. M. May, Infectious diseases of humans: dynamics and control, Oxford university press, 1992.

[16] L. C. Anderson, E. S. Loker, H. J. Wearing, Modeling schistosomiasis transmission: the importance of snail population structure, Parasites & vectors 14 (1) (2021) 1–14.

[17] G. M. Niemann, F. A. Lewis, Schistosoma mansoni: influence of biomphalaria glabrata size on susceptibility to infection and resultant cercarial production, Experimental Parasitology 70 (3) (1990) 286–292.

[18] Z. Feng, C.-C. Li, F. A. Milner, Schistosomiasis models with density dependence and age of infection in snail dynamics, Mathematical Biosciences 177 (2002) 271–286.

[19] J. H. Cummings, S. A. Bingham, K. W. Heaton, M. A. Eastwood, Fecal weight, colon cancer risk, and dietary intake of nonstarch polysaccharides (dietary fiber), Gastroenterology 103 (6) (1992) 1783–1789.

[20] T. D. Mangal, S. Paterson, A. Fenton, Predicting the impact of long-term temperature changes on the epidemiology and control of schistosomiasis: a mechanistic model, PLoS one 3 (1) (2008) e1438.

[21] O. Diekmann, J. A. P. Heesterbeek, J. A. Metz, On the definition and the computation of the basic reproduction ratio r 0 in models for infectious diseases in heterogeneous populations, Journal of mathematical biology 28 (4) (1990) 365–382.
